# Epithelial to mesenchymal transition (EMT) is associated with attenuation of succinate dehydrogenase (SDH) in breast cancer through reduced expression of *SDHC*

**DOI:** 10.1186/s40170-019-0197-8

**Published:** 2019-06-01

**Authors:** Gro V. Røsland, Sissel E. Dyrstad, Deusdedit Tusubira, Reham Helwa, Tuan Zea Tan, Maria L. Lotsberg, Ina K. N. Pettersen, Anna Berg, Charlotte Kindt, Fredrik Hoel, Kirstine Jacobsen, Ari J. Arason, Agnete S. T. Engelsen, Henrik J. Ditzel, Per E. Lønning, Camilla Krakstad, Jean P. Thiery, James B. Lorens, Stian Knappskog, Karl J. Tronstad

**Affiliations:** 1 Department of Biomedicine, University of Bergen, Bergen, Norway; 2 Department of Clinical Science, University of Bergen, Bergen, Norway; 3 Faculty of Science, Ain Shams University, Cairo, Egypt; 4 Cancer Science Institute of Singapore, National University of Singapore, Singapore, Singapore; 5 Department of Pathology, Haukeland University Hospital, Bergen, Norway; 6 Department of Gynecology and Obstetrics, Haukeland University Hospital, Bergen, Norway; 7 Department of Molecular Medicine, University of Southern Denmark, Odense, Denmark; 8 Biomedical Center, University of Iceland, Reykjavík, Iceland; 9 Centre for Cancer Biomarkers (CCBIO), Department of Clinical Medicine, Faculty of Medicine and Dentistry, The University of Bergen, Bergen, Norway; 10 Department of Oncology, Odense University Hospital, 5000 Odense, Denmark; 11 Department of Oncology, Haukeland University Hospital, Bergen, Norway; 12 Biomedical Department of Biochemistry, Yong Loo Lin School of Medicine, National University of Singapore, Singapore, Singapore; 13 Inserm Unit 1186 Comprehensive Cancer Center Institut Gustave Roussy, Villejuif, France; 14 Institute of Molecular and Cell Biology, A-STAR, Singapore, Singapore

**Keywords:** Cell plasticity, Cell metabolism, Mitochondria, SDH, Breast cancer

## Abstract

**Background:**

Epithelial to mesenchymal transition (EMT) is a well-characterized process of cell plasticity that may involve metabolic rewiring. In cancer, EMT is associated with malignant progression, tumor heterogeneity, and therapy resistance. In this study, we investigated the role of succinate dehydrogenase (SDH) as a potential key regulator of EMT.

**Methods:**

Associations between SDH subunits and EMT were explored in gene expression data from breast cancer patient cohorts, followed by in-depth studies of SDH suppression as a potential mediator of EMT in cultured cells.

**Results:**

We found an overall inverse association between EMT and the SDH subunit C (SDHC) when analyzing gene expression in breast tumors. This was particularly evident in carcinomas of basal-like molecular subtype compared to non-basal-like tumors, and a low *SDHC* expression level tended to have a prognostic impact in those patients. Studies in cultured cells revealed that EMT was induced by SDH inhibition through SDHC CRISPR/Cas9 knockdown or by the enzymatic inhibitor malonate. Conversely, overexpression of EMT-promoting transcription factors TWIST and SNAI2 caused decreased levels of SDHB and C and reduced rates of SDH-linked mitochondrial respiration. Cells overexpressing TWIST had reduced mitochondrial mass, and the organelles were thinner and more fragmented compared to controls.

**Conclusions:**

Our findings suggest that downregulation of SDHC promotes EMT and that this is accompanied by structural remodeling of the mitochondrial organelles. This may confer survival benefits upon exposure to hostile microenvironment including oxidative stress and hypoxia during cancer progression.

**Electronic supplementary material:**

The online version of this article (10.1186/s40170-019-0197-8) contains supplementary material, which is available to authorized users.

## Introduction

Epithelial to mesenchymal transition (EMT) provides a useful mechanistic framework for studying the regulation and dynamics of cell fate transitions (i.e., cell plasticity) central to developmental and cancer cell biology [1–3]. Events involving downregulation or dysfunction of mitochondrial enzymes have been linked to EMT, but the potential role of mitochondrial remodeling as part of the EMT program has not yet been evaluated through systematic studies of mitochondrial physiology [4].

EMT is a reversible transdifferentiation program whereby epithelial cells convert into migratory mesenchymal cells with enhanced cell survival attributes [1, 2]. EMT is recognized by a loss of epithelial markers such as cytokeratins and E-cadherin, followed by a concomitant increase in mesenchymal markers such as N-cadherin and vimentin [5]. In cancer development, this is associated with therapy resistance and poor clinical outcome [6]. The cellular processes of EMT are orchestrated by several key transcription factors (e.g., TWIST, SNAI1, SNAI2, ZEB1/2) that act in concert with epigenetic mechanisms and post-translational protein modifications to coordinate the cellular alterations [1]. Application of gene expression signatures combining multiple EMT-linked genes has proven useful to evaluate EMT as a contributing factor in tumor development in human cancers [7].

Cellular metabolism provides the energy and building blocks required for cell function and growth and is regulated in close relation to changes in the physiological state of the cell and in the microenvironment [8]. To this end, mitochondrial reprogramming has been shown to be of significance in oncogenic events [9, 10]. Several oncometabolites recognized as drivers of tumor development and progression have been identified, including fumarate, D-2-hydroxyglutarate (D-2HG), and succinate [11]. Such metabolites have been found to have causative influence in cancers with genetic deficiencies in associated enzymes, including fumarate hydratase (FH) [12], isocitrate dehydrogenase 1 (IDH1) [8], and succinate dehydrogenase (SDH) [13]. Mutations (germline) in SDH subunits have been linked to familial paraganglioma syndromes, pheochromocytomas (PGL/PCC), renal cell carcinomas (RCC), and gastrointestinal stromal tumors (GISTs) [14], both as predisposing and prognostic factors [13]. Thus, in PGL/PCC, GIST, and RCC, SDH is classified as a tumor suppressor [13, 15–18].

The SDH complex, also referred to as respiratory complex II in the mitochondrial electron transport chain, is composed of four subunits (SDHA, SDHB, SDHC, and SDHD). It has a central role in energy metabolism, as it directly links the tricarboxylic acid cycle (TCA-cycle) to the respiratory machinery [19]. SDHA and SDHB are hydrophilic subunits and form the catalytic unit of the complex, whereas SDHC and SDHD represent the hydrophobic membrane-bound part of the complex. SDH genes can act as classic tumor suppressor genes, as the mutated alleles often are inherited in a heterozygous manner, and the respective wild-type allele is lost in tumors [9]. Mutations in or downregulation of the SDHB subunit have previously been associated with TGFβ-induced EMT in cancer cells [20–22]. In a previous study investigating breast cancer, the protein expression level of SDHA and B was lost in 3% of the samples [23]. Such effects may indicate that metabolic rewiring could be a facilitating feature for cell plasticity, as it also has been linked to cell state transitions such as differentiation, senescence, and oncogenic transformation [4, 24–27]. In summary, there are several observations supporting that genetic defects in mitochondrial enzymes may affect features of EMT [4, 13, 28, 29]. However, the potential role of metabolic rewiring as a more general driving force of cellular plasticity in human tumors remains poorly explored.

In this study, we present gene expression analysis of human breast cancer samples, correlating the level of SDH subunits to the levels of EMT-related genes. We show that reduced expression of *SDHC* was particularly associated with EMT in the breast cancer cohorts of this study, especially the ductal- and basal-like subgroups. In subsequent cell studies, we found a bilateral causative relationship between SDH attenuation and EMT induction, which involved significant changes in mitochondrial morphofunctional properties.

## Methods

### Gene expression analysis of human breast cancer samples

We investigated the association between EMT and SDH genes in a breast cancer patient cohort obtained from the Haukeland University Hospital (*n* = 204) [30], as well as an Affymetrix breast cancer meta-cohort (*n* = 3992) [7]. In this study, we used two distinct signatures, one generic comprising 315 genes related to EMT in various tissues (EMT315 signature) [7], and the other consisting of 8 genes of particular relevance for EMT in breast cancer (EMT8 signature). The EMT8 signature was designed based on a previously described 5-gene signature (*CDH1*, *CTNNB1*, *CTNNA2*, *CDH2*, *CDH3*) [31], which we extended with *KRT19*, an established marker for breast cancer cells, and *SNAI2* and *TWIST* due to their role as determinants of EMT in breast cancer metastasis and invasion. The correlation between the two different EMT signature scores was strong in our study cohorts (for the meta-cohort *Rho* = 0.674, *p* < 0.0001 and for the *n* = 204 cohort *Rho* = 0.6651, *p* < 0.0001). Further details about the gene expression analysis (GEA) are provided in Additional file 1: Supplemental methods.

### Cell models

The breast epithelial cell line MCF10A and the breast cancer cell line MCF7 (both from ATCC, Manassas, VA) were cultured according to conventional procedures (further described in Additional file 1: Supplemental methods).

### Overexpression of EMT-linked transcription factors

Stable modified MCF10A subclones overexpressing *TWIST* or *SNAI2* were established by retroviral transduction, as described previously [32], and termed MCF10A/TWIST and MCF10/SNAI2, respectively. The plasmid constructs used are previously described [33]. The cells were exposed to the virus for 2 × 8 h, interrupted by 8-h incubation in standard medium. In addition, a control subclone was prepared by insertion of the empty vector, which contained the gene for GFP (MCF10A/GFP). Transduction positive cells were sorted by FACS using the GFP marker.

### CRISPR/Cas9 in vitro gene editing of *SDHC* and *SDHD*

MCF7 cells with heterozygous knockdown of *SDHC* (MCF7 *SDHC*^*+/−*^) was obtained by introducing a frameshift deletion within the coding region (exon 3) of the gene. Twenty nucleotides gRNA targeting *SDHC* were designed (ATAGTAATGTGGGGAGACAG) using the Benchling online tool (www.benchling.com). The oligo-nucleotide sequences were synthesized with the suitable overhangs for plasmid insertion (CACCGATAGTAATGTGGGGAGACAG and AAACCTGTCTCCCCACATTACTATC), before insertion into the pX458SpCas9 plasmid (Addgene, Waltertown, MA, USA), which had been modified to increase the fidelity of Cas9, (according to [34], kindly provided by Ole M. Seternes). The primers were phosphorylated and annealed using T4 PNK (NEB), followed by digestion/ligation into the plasmid, utilizing Golden Gate reaction using BbsI enzyme (NEB) and T7 ligase (NEB). The gRNA inserts were further sequenced to confirm the correct insertion using the U6 primer (GATACAAGGCTGTTAGAGAGATAATT). The cells were transfected with the gRNA containing construct using Lipofectamine LTX (Invitrogen, Carlsbad, CA, USA) for 5 days. Subsequently, cells were sorted into a 96-well plate (one cell per well) based on GFP expression from the vector, using Sony SH800S cell sorter. Upon colony formation in the wells, DNA was purified from each clonal colony and the targeted region was amplified by PCR and sequenced using forward primer CTCGGCCTCCCAAAGAGCTGAGATTA and reverse primer CTCATCTACATAGCAGTATTTTGGTTGAGTAA. The PCR products revealing deletion(s) were further inserted into (vector) by TOPO TA cloning and subject to re-sequencing, in order to confirm that mutation was introduced.

### mRNA expression analysis by quantitative polymerase chain reaction

Total RNA was isolated from cell pellets using the RNeasy MINI KIT (74104, Qiagen, Venlo, Netherlands). cDNA was synthesized using the High-Capacity cDNA Reverse Transcription Kit (4368813, Thermo Fisher Scientific, Waltham, MA) and Biorad MJ Mini Thermal Cycler (Hercules, CA, USA). The quantitative polymerase chain reaction (qPCR) was performed using the Light Cycler 480 system (Roche, Basel, Germany) and the Light Cycler 480 Probes Master reaction mix (Cat# 04887301001, Roche). The gene-specific probes used are listed in Additional file 2: Table S3. The ∆∆ct method was used for calculating fold change in gene expression relative to the control sample.

### Mitochondrial DNA quantification

Total DNA was purified from cell pellets using the DNeasy blood and tissue kit from Qiagen (69504, DNeasy Blood and tissue kit, Qiagen). Taqman probe/primer mixtures for mitochondrial NADH dehydrogenase 1 (mitochondrial DNA (mtDNA) gene; Hs02596873_s1 MTND1) and eukaryotic 18 s rRNA (nuclear gene; 4333760F, Applied Biosystems, CA, USA) were used. Following quantification by qPCR, the amount of mtDNA relative to nuclear DNA was calculated as the ratio between the levels of MTND1 and 18 s RNA using the ΔΔct method [32].

### Western blot analysis

Cells were scraped and lysed in RIPA lysis buffer (sc24948, Santa Cruz Biotechnology, Dallas, TX). Protein concentration of the lysed samples were measured by the Pierce® BCA Protein Assay Kit (Thermo Fisher Scientific). Electrophoresis was done using premade Biorad stain-free gels (Biorad Mini-PROTEAN 3 Cell), and the protein was transferred to polyvinylidene fluoride PVDF membranes (GE Healthcare, Little Chalfont, U.K) by BioradTurbo Transfer System. Before stained with respective antibodies (Additional file 2: Table S3), total protein was assessed by imaging on ChemiDocTM XRS+ with Image Lab Software (Biorad).

### Spheroid and scratch-wound assays

For measurement of spheroid formation capacity, Geltrex LDEV-free (Thermo Fisher Scientific) was used as a gel matrix. In a 12-well plate, 350 μl/well of Geltrex was casted and solidified at 37 °C for 30 min. Twenty-five thousand cells were suspended in 500 μl of assay medium (2% of geltrex in medium) and added to the solidified matrix. Cells were incubated at 37 °C and cell growth and colonies were observed for 3–7 days. For analysis of spheroid stability and growth, centrifugation-assisted spheroid formation was performed by transferring the cells (5000 cells/well) to a 96-well u-bottom ultralow attachment plate (Corning, Thermo Fisher Scientific), followed by centrifugation for 15 min at 300 rcf (room temperature). The Incucyte ZOOM 2016B (EssenBioscience Ltd., UK) was used for time-lapse imaging of the spheroids and for scratch-wound assay. For MCF7 spheroids, the area was calculated from the average radius retrieved from measuring two perpendicular diameters (Image Pro Software version 7.0, Media Cybernetics, Inc., Washington, USA). Ten spheroids were measured in each group. For the scratch-wound assay, cells were plated at 45 k cells/well (IncuCyte ImageLock Plates cat #4397) for an optimal 80–90% confluency and incubated over-night. Just prior to the time-lapse imaging sequence, scratch wound was made to the monolayer using the wound maker (IncuCyte Cell Migration Kit, cat# 4493). The cultures were imaged using the Incucyte ZOOM 2016B or by phase contrast microscopy of monolayers fixed in methanol with crystal violet. The percent wound closing after 24 h relative to start was measured using the IncuCyte scratch-wound cell migration software module (Cat# 9600-0012), or by phase contrast microscopy with ocular micrometer, measuring the gap distance at a fixed location.

### Mitochondrial respiration

Oxygen consumption rate (OCR) was measured using the Seahorse XFe96 Analyzer (Agilent, Santa Clara, CA), according to the manufacturer’s protocols and previous descriptions [32, 35]. All materials were from Sigma-Aldrich (St. Louis, MO) unless otherwise stated. For analysis of SDH-linked activity, the cells were permeabilized to facilitate cellular uptake of succinate and ADP, by adding the Seahorse XF plasma membrane permeabilizer (PMP) (Agilent), as indicated. The concentration of PMP and metabolic modulators (uncoupler, inhibitors) were optimized for each cell type. The data were normalized to cell number using Hoechst 33342 (Thermo Fisher Scientific) or protein content (Pierce® BCA Protein Assay Kit, Thermo Fisher Scientific). Further details are provided in Additional file 1: Supplemental methods.

### Flow cytometric G0–G1 separation by Pyronin Y and Hoechst 33258 staining

Cell pellets (1 mill cells) were treated with 50 μg/ml Hoechst 33342 (Thermo Fisher Scientific) for 1 h in 37 °C. After washing, the cells were stained with 1 μg/ml Pyronin Y (Sigma-Aldrich) for 30 min in 37 °C. Cells were washed and filtered before flow cytometric analysis. Pyronin Y was detected at ca 570 nM and Hoechst was detected at 405 nm at the Fortessa LSR (BD Biosciences, San Jose, CA). Analysis was performed in FlowJo software.

### Immunocytochemistry

Cells were plated on cover slips in 24-well culture plates (10,000 cells/well) and left until they reached 70% confluency. The cells were then fixed in 3.7% PFA, permeabilized with TBS-T, and stained with primary antibodies for E-cadherin (cat # 14472, Cell Signaling, Leiden, Netherlands) and vimentin (AB92547, Abcam, UK), and diluted 1:100 in TBS-T with 0.5% BSA. Alexa 594 anti-mouse and Alexa-647 anti-rabbit were used as secondary antibodies. The cells were thereafter stained with 1:40 Phalloidin AF555 (a34055, Thermo Fisher Scientific) according to the manufacturer’s instruction, before mounting with Prolong Dimond with Dapi (Thermo Fisher Scientific). Images were acquired on a Leica TC2 SP8 STED 3X with HC PL APO CS2 lasers using the 100 × 1.4 NA oil objective.

### Confocal microscopy and three-dimensional image analysis of mitochondria

Mitochondria were stained using immunocytochemistry (ICC) (as described above) with primary antibodies against TOM20 (FL145 Santa Cruz Biotech, Dallas, Texas; 1:100); and ATPB (AB5452 Abcam, Cambridge, UK; 1:500). Imaging was performed by confocal microscopy (Leica TCS S5 microscope, Leica microsystems, Wetzlar, Germany). Image processing, three-dimensional (3D)-modeling and quantitative analysis of mitochondrial structures were performed using the Image-Pro Plus software (version 7.0) (Media Cybernetics), as described previously [32, 36]. Further details are provided in Additional file 1: Supplemental methods.

## Results

### Association between EMT and reduced *SDHC* expression in human breast cancer

**Fig. 1:**
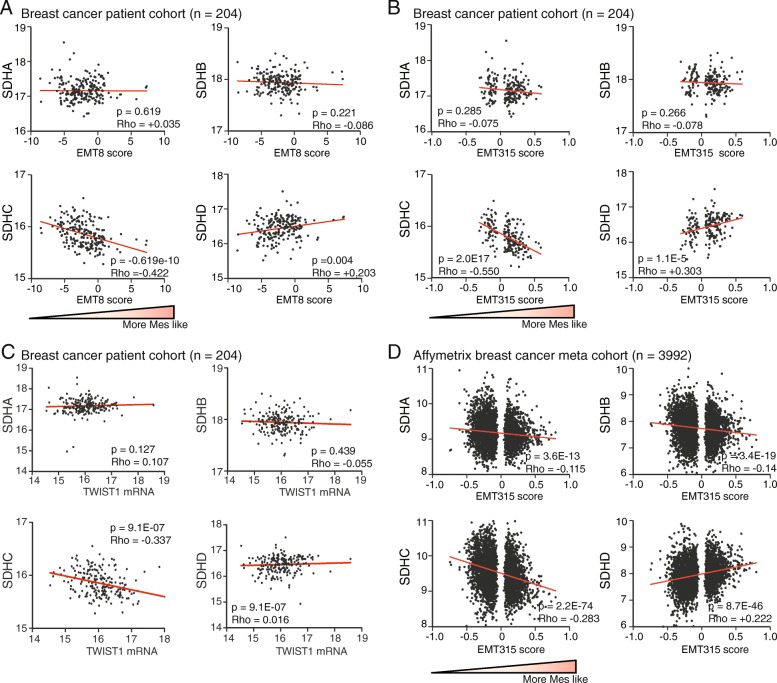
Association between SDH and EMT in gene expression data from breast cancer patient cohorts. Gene expression (mRNA) correlation analysis (Spearman) between SDH subunits and EMT signature in datasets from breast cancer patients. **a** Breast cancer patient cohort (*n* = 204), using the EMT8 signature (8 genes). **b** Breast cancer patient cohort (*n* = 204), using the EMT315 signature (315 genes). **c** Breast cancer patient cohort (*n* = 204), correlation with TWIST1 expression. **d** Affymetrix breast cancer patient meta cohort (*n* = 3992), relative to the EMT315 signature. The gene expression data are displayed with relative arbitrary units

We investigated the association between EMT and the different SDH subunits in human breast tumors, based on gene expression in a patient cohort obtained from the Haukeland University Hospital (*n* = 204) [30] and an Affymetrix breast cancer meta-cohort (*n* = 3992) [7]. Using both a breast cancer-directed 8-gene signature (EMT8) and a generic 315-gene signature (EMT315), EMT was found to be particularly associated with reduced *SDHC* expression in the *n* = 204 cohort, with a *Rho* value of − 0.422 (*p* < 0.0001) using the EMT8 signature (Fig. 1a) and *Rho* value of − 0.55 (*p* < 0.0001) using the EMT315 signature (Fig. 1b). *SDHA* and *SDHB* expression were not associated with EMT, whereas *SDHD* demonstrated a positive relationship upon using the EMT315 signature (*Rho* = 0.303, *p* < 0.0001) (Fig. 1b). We also investigated if the expression of the SDH subunits was specifically associated with the central EMT-linked transcription factors TWIST and SNAI2. The analysis indicated that *SDHC* was inversely correlated to both *TWIST1* (*Rho* = − 0.337, *p* < 0.0001) (Fig. 1c) and *SNAI2* (*Rho* = 0.27, *p* < 0.0001), whereas *SDHD* showed a positive correlation to *SNAI2* (*Rho* = 0.328, *p* < 0.0001) (Additional file 2: Figure S1A). Neither *SDHA*, *SDHB*, nor *SDHD* showed associations to *TWIST1*. To verify these results, we applied the EMT315 signature on the Affymetrix breast cancer patient meta-cohort (*n* = 3992). In concordance with our previous observations in the *n* = 204 cohort, there was an inverse association between EMT and *SDHC* (*Rho* = − 0.283, *p* < 0.0001) (Fig. 1d). In support, we found a similar inverse correlation between EMT315 and *SDHC* gene expression in a breast invasive carcinoma cohort (*Rho* = − 0.337, *p* < 0.0001) as well as in breast cancer cell lines (*Rho = −* 0*.*517, *p* < 0.0001) in TCGA RNA-seq data (Additional file 2: Table S1). Correlation analysis between the individual SDH subunits in the *n* = 204 cohort generally returned statistically significant associations between subunit pairs, although with relatively low *Rho*-value (Additional file 2: Figure S1B). This suggests some level of co-regulation of the expression of the individual subunits, as would be expected since the resulting proteins belong to the same enzyme complex. In summary, these data linked EMT to *SDHC* suppression in breast cancer and suggested that TWIST and SNAI2, two of the master promoters of EMT, could be involved.

### Low *SDHC* expression is associated with a poor prognosis in basal-like tumors

**Fig. 2:**
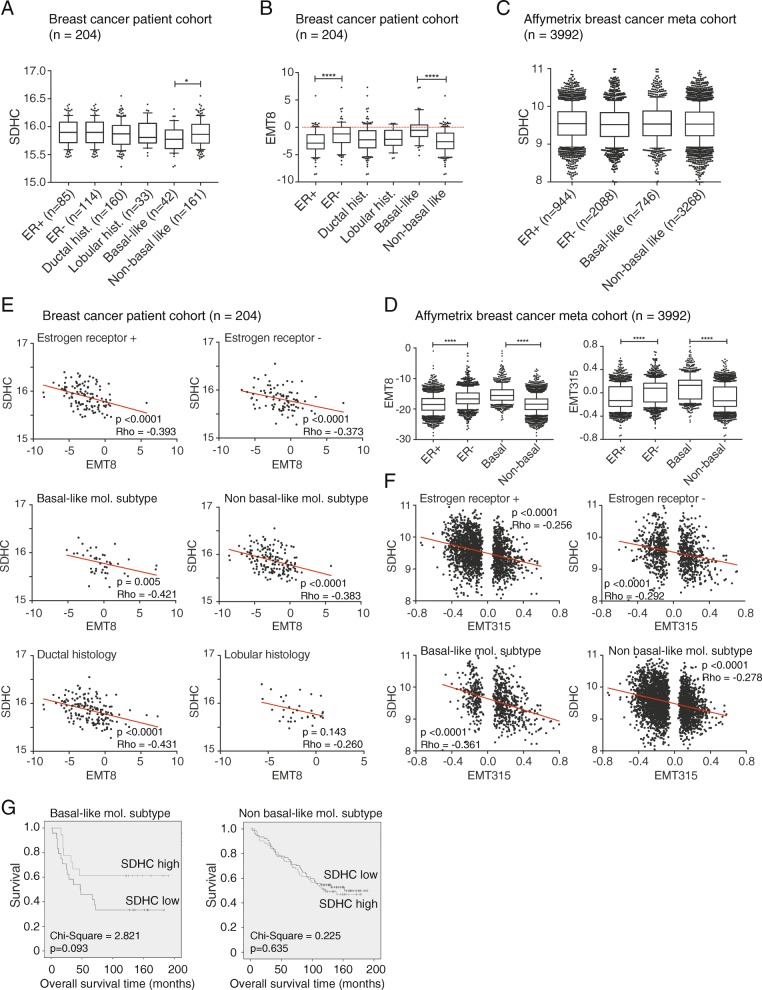
*SDHC* gene expression in subgroups of breast cancer. The two cohorts included in the study was subdivided based on molecular characteristics such as estrogen receptor positive and negative (ER+ and ER−) and basal- and non-basal phenotype. Claudin-low and triple negative subgroups were included in the basal category. In addition, the breast cancer patient cohort (*n* = 204) was subgrouped based on histology, i.e., into ducal and lobular characteristics. **a** mRNA expression of *SDHC* and **b** EMT8 signature were assessed for the distinct subgroups in the *n* = 204 cohort. **c** mRNA expression of *SDHC* and **d** EMT8 and EMT315 signatures were determined for the subgroups in the *n* = 3992 Affymetrix meta cohort. **e** Spearman correlation analysis for *SDHC* expression relative to EMT8 signature for subgroups of the breast cancer cohort (*n* = 204) and **f** the Affymetrix meta cohort. **g** Kaplan-Meier survival plots for basal- (*n* = 42) and non-basal-like (*n* = 161) breast carcinoma of the breast cancer cohort. The gene expression data are displayed with relative arbitrary units

Next, we investigated the relationship between EMT and *SDHC* when the cohort was divided into molecular subgroups. When we looked at the relative levels of SDHC mRNA, we found no difference between the *n* = 204 cohort tumors classified as either ER+ and ER−, or as ductal and lobular carcinoma (Fig. 2a). Noteworthy, we found significantly lower *SDHC* expression in basal-like compared to non-basal tumors, and this coincided with significantly higher EMT8 score (Fig. 2b). A higher EMT8 score was seen in ER− tumors compared to ER+, but there was no difference between the histological ductal and lobular types. This is in agreement with the known phenotypic differences between basal-like and other breast tumor subtypes. The higher EMT status in ER− vs ER+ tumors and basal-like vs non-basal-like tumors were further supported in the Affymetrix meta-cohort, using either of the EMT signatures (Fig. 2d); however, the overall *SDHC* expression did not differ between the subgroups of this cohort (Fig. 2c). Histological subclassification (i.e., ductal vs lobular carcinoma) was not available for the Affymetrix meta-cohort. Correlation analysis was performed to evaluate the relationship between *SDHC* expression and EMT status within each breast cancer subgroup. The SDHC mRNA level was inversely associated with the EMT8 score in each subgroup of the *n* = 204 cohort (with *Rho* between − 0.431 and − 0.373), except for lobular breast cancer (Fig. 2e). In support, we found an inverse association between *SDHC* expression and EMT315 score in the subgroups of the Affymetrix meta-cohort, somehow stronger in basal-like tumors (*Rho* = − 0.361, *p* < 0.0001) compared to the non-basal-like tumors (with *Rho* between − 0.292 and − 0.256) (Fig. 2f). Interestingly, low *SDHC* expression tended to be associated with poorer survival in patients with basal-like tumors, compared to patients with a high level of *SDHC* (chi-square = 2.821, *p* = 0.093) (Fig. 2g). This trend was not seen in patients with non-basal like tumors.

### SDH attenuation by *SDHC* knockdown induces EMT in breast cancer cells (MCF7)

**Fig. 3:**
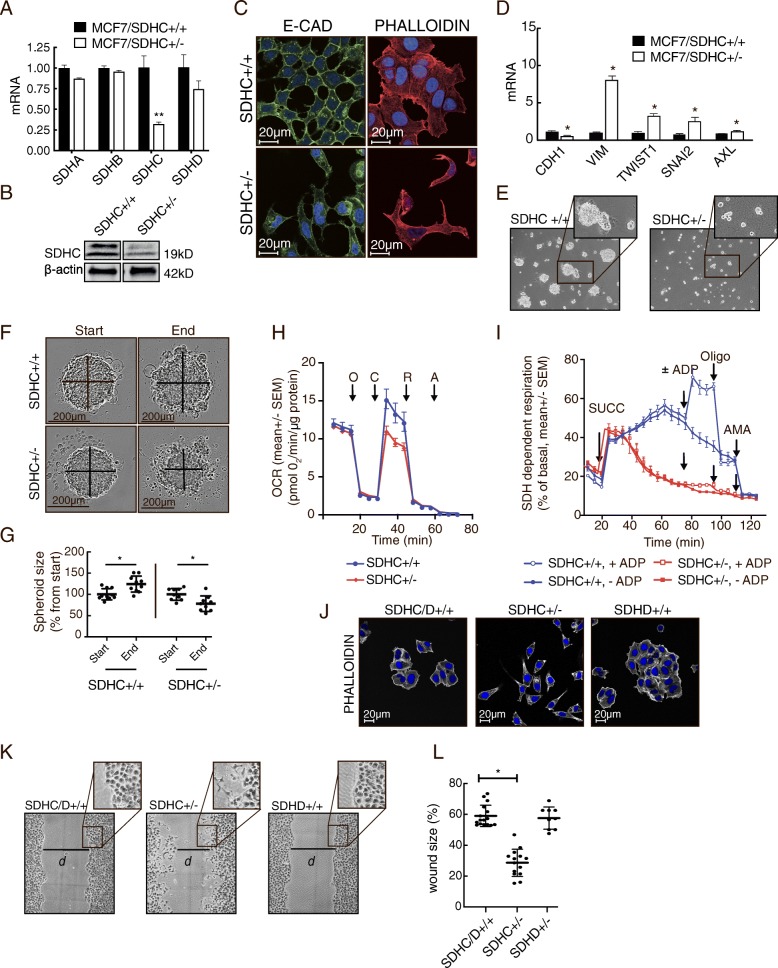
Induction of EMT in MCF7 upon *SDHC* knockdown. Parental MCF7 cells (MCF7 *SDHC*^*+/+*^) were modified by CRISPR/Cas9 editing to knock down the expression of SDHC (MCF7 *SDHC*^*+/−*^). **a** *SDHA-D* mRNA was analyzed by qPCR. **b** SDHC protein expression was analyzed by western blotting. **c** Confocal microscopy was performed to evaluate E-cad (immuno-stained, green) expression level and cell morphology (F-actin stained by phalloidin, red). **d** mRNA expression of the EMT markers E-cad (CDH1), vimentin (VIM), TWIST1, SNAI2, and Axl. **e** Spheroid formation (anchorage-independent) was evaluated after seeding the cells in wells with low surface adherence. **f** Spheroid growth and stability was assessed after centrifugation-aided spheroid formation. The spheroid size was measured after 48 h in culture. **g** The diagram shows statistical data from the experiment described in (**f**). **h** Mitochondrial respiratory rates were measured in MCF7 *SDHC*^*+/+*^ and MCF7 *SDHC*^*+/−*^ cultures, with glucose, pyruvate, and glutamine provided as the major fuels. Oxygen consumption rate (OCR) was monitored upon sequential additions of oligomycin (O, 3 μM), CCCP (C, 0.75 μM), rotenone (R, 1 μM), and antimycin A (A, 1 μM) as indicated, to assess specific properties of mitochondrial respiration. **i** For measurement of SDH-dependent mitochondrial respiration, the cells were permeabilized (with PMP) and rotenone was added prior to analysis in restricted assay medium (MAS). Succinate (SUCC, 10 mM), ADP (4 mM), oligomycin (OLIGO, 3 μM), and antimycin A (AMA, 1 μM) were added sequentially as indicated. **j** Fluorescence microscopy was performed to compare cell morphology (F-actin stained by phalloidin, white) in MCF7 *SDHD/C*^*+/+*^, MCF7 *SDHC*^*+/−*^_,_ and MCF7 *SDHD*^*+/−*^ cultures. **k** Scratch-wound assay comparing MCF7 *SDHD/C*^*+/+*^, MCF7 *SDHC*^*+/−*^_,_ and MCF7 *SDHD*^*+/−*^ cells. The images were taken 24 h after the scratch was made. **l** In the experiment described in (**k**), we measured scratch size as gap distance (*d*) at a fixed position, after 24 h, and calculated the results relative to the initial scratch size. Each dot represents separate wells. Data are shown as mean ± SD for (**a**), (**d**), (**g**), and (**l)** and mean ± SEM for (**h)** and (**i)**. Student’s *t* test was used for statistical analysis. *p < 0.01; ns, not significant

The initial gene expression analysis in human breast tumors and cell lines convincingly suggested that EMT is associated with reduced *SDHC* expression. To explore the impact of reduced *SDHC* expression on EMT-related features, we knocked down this gene in MCF7 breast cancer cells, using the CRISPR/Cas9 system. Successful heterozygous CRISPR/Cas9 editing of *SDHC* (MCF7 *SDHC*^*+/−*^) was confirmed by sequencing (Additional file 2: Figure S2), and the resulting reduction in SDHC mRNA and protein was verified (Fig. 3a and b). Immunostaining and fluorescence microscopy indicated reduced protein levels of E-cadherin in MCF7 *SDHC*^*+/−*^ compared to MCF7 *SDHC*^*+/+*^cells, and F-actin staining with phalloidin revealed a concordant transition from epithelial to mesenchymal-like cell morphology (Fig. 3c). The knockdown of *SDHC* was also accompanied by marker expression consistent with induction of EMT, i.e, E-cadherin (*CDH1*) was downregulated and vimentin (*VIM*), *SNAI2*, *TWIST*, and *AXL* [33] were upregulated (Fig. 3d). The level of N-cadherin (*CDH2*) mRNA was undetectable in both MCF7 *SDHC*^*+/+*^ and MCF7 *SDHC*^*+/−*^ cells. Consistent alterations in morphological phenotype were also visualized by contrast enhancement microscopy during the course of these experiments. The MCF7 *SDHC*^*+/−*^ cells demonstrated reduced capacity to form spheroids in plates with low surface adherence when compared to MCF7 *SDHC*^*+/+*^ cells (Fig. 3e). Further, following centrifugation-aided spheroid formation, the MCF7 *SDHC*^*+/−*^ spheroids decreased in size, whereas the MCF7 *SDHC*^*+/+*^ spheroids grew significantly (Fig. 3f and g). This reduced growth and stability of the multicellular spheroids are consistent with a mesenchymal phenotype, as is the loss of cell-cell adherence observed near the periphery of the MCF7 *SDHC*^*+/−*^ spheroids.

In order to study effects of *SDHC* knockdown on mitochondrial respiration, we measured the oxygen consumption rate (OCR) under normal cell culture conditions (DMEM medium) with glucose, pyruvate, and glutamine as the major fuels and then under conditions specifically composed to access changes in SDH function. In the presence of glucose, pyruvate, and glutamine as the major oxidative substrates, the MCF7 *SDHC*^*+/−*^ cells demonstrated normal basal respiratory rate; however, they had significantly reduced uncoupled respiratory capacity after addition of oligomycin and CCCP (Fig. 3h). To investigate succinate-dependent mitochondrial respiration, OCR was measured in permeabilized cells, in the presence of the complex I inhibitor, rotenone, and with succinate as the only oxidation fuel (Fig. 3i). After adding succinate, the OCR increased immediately and continued to rise in the MCF7 *SDHC*^*+/+*^ cultures. In contrast, succinate caused only a transient OCR induction in the MCF7 *SDHC*^*+/−*^ cultures. Furthermore, while OCR increased after addition of ADP in MCF7 *SDHC*^*+/+*^ cultures, there was no effect of ADP for MCF7 *SDHC*^*+/−*^. The increased OCR after addition of ADP, and the subsequent inhibition by the ATP synthase inhibitor oligomycin, confirms that this SDH-linked respiration was coupled to ATP production through oxidative phosphorylation (OXHOS) in the MCF7 *SDHC*^*+/+*^ cells. The absence of such ADP-linked effects in the MCF7 *SDHC*^*+/−*^ cells indicates that these cells were unable to utilize succinate to fuel ATP production. In summary, the MCF7 *SDHC*^*+/−*^ cells were incapable of maintaining succinate-driven mitochondrial respiration and OXPHOS under these conditions, consistent with an attenuation of SDH activity.

Further, properties of migration were investigated in a scratch wound experiment, where we also included MCF7 *SDHD*^*+/−*^ cells (sequencing data in Additional file 2: Figure S2B), as SDHD demonstrated a different relationship with EMT compared to SDHC in the previous tumor gene expression analysis. Similar to the control MCF7 *SDHC*/D^+/+^ cells (parental), and in contrast to the mesenchymal-like MCF7 *SDHC*^+/−^ cells, the MCF7 *SDHD*^+/−^ cells had an epithelial morphology (Fig. 3j). The scratch-wound study clearly showed that MCF7 *SDHC*^*+/−*^ cells had significantly higher wound healing capacity compared to MCF7 *SDHC/D*^*+/+*^ and MCF7 *SDHD*^*+/−*^ cells, as evident by a significantly smaller gap distance 24 h after the wound was made (Fig. 3k and l).

### SDH enzyme inhibition triggers EMT

**Fig. 4:**
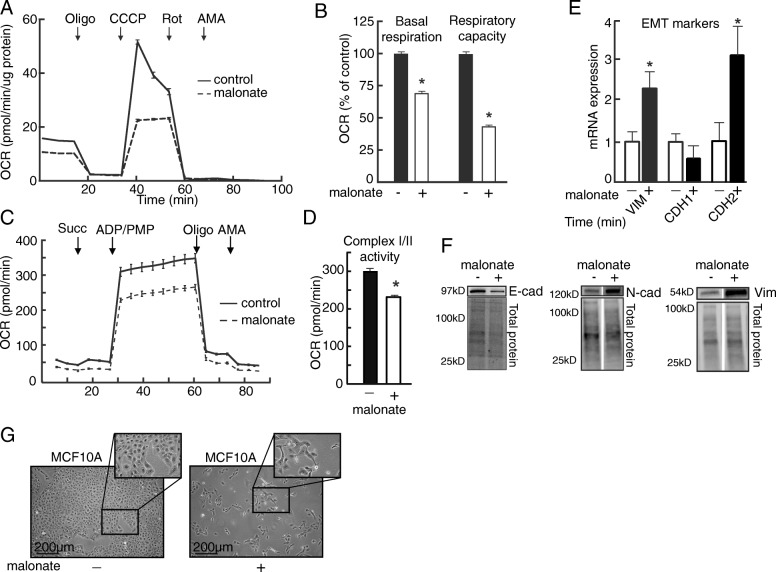
SDH enzyme inhibition causes induction of EMT. We investigated if SDH enzyme inhibition causes induction of EMT by treating MCF10A cells with the SDH inhibitor malonate (25 mM) for 3 days. **a** Conventional analysis of mitochondrial respiratory function by measuring oxygen consumption rate (OCR) in DMEM medium in malonate treated MCF10A cells. **b** The diagram shows statistical data from the experiment described in (**a**). **c** SDH-linked respiration was assessed with succinate (Succ) as the only provided substrate, following the addition of cell permeabilizing agent (PMP) and ADP. Oligomycin (Oligo) and antimycin A (AMA) was added to control mitochondrial integrity and background activity, respectively. **d** The diagram shows statistical data from the experiment described in (**c**). **e** mRNA and **f** protein expression of epithelial (E-cadherin (*CDH1*)) and mesenchymal (N-cadherin, (*CDH2*); vimentin, (*VIM*)) markers. **g** Phase contrast microscopy. Student’s *t* test was used for statistical analysis. Data are shown as mean ± SEM for (**a**)–(**d**) and mean ± SD for (**e**). *p < 0.01

The results so far suggested that EMT is associated with downregulation of SDH and that defective function of this enzyme may be a causative factor for EMT in tumors. To investigate if this link between EMT and SDH has a general relevance also in non-tumorigenic cells, we studied the effects of the competitive succinate dehydrogenase enzymatic inhibitor malonate in the human mammary epithelial cell line MCF10A. Treatment with malonate for 3 days significantly reduced basal respiration and uncoupled respiratory capacity (Fig. 4a and b). Further, malonate treatment was confirmed to inhibit SDH by reducing SDH-linked respiration measured in permeabilized cells in the presence of succinate and ADP (Fig. 4c and d). In both of these experiments, the normal response to the addition of oligomycin, and subsequently ADP or uncoupler, confirmed that the integrity of the OXPHOS system remained intact upon malonate treatment. Importantly, the malonate treatment caused increased expression of vimentin and N-cadherin and reduced expression of E-cadherin, both on the level of mRNA (Fig. 4e) and on protein (Fig. 4f). This typical marker profile of EMT was consistent with the consequent change in cellular morphology (Fig. 4g). These data support that inhibition of SDH enzyme activity may constitute an inherent trigger of the EMT program.

### Overexpression of EMT-linked transcription factors leads to attenuation of SDH

**Fig. 5:**
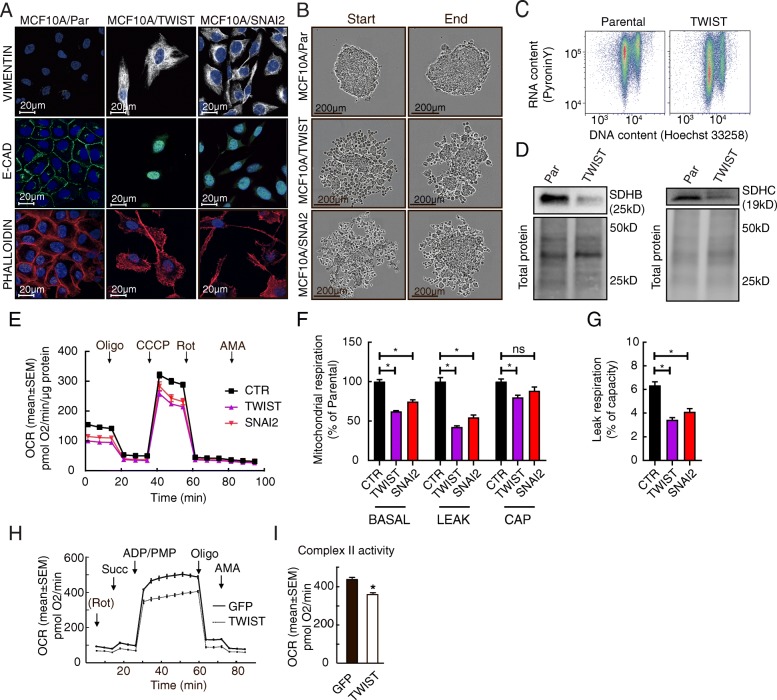
Induction of EMT in MCF10A cells overexpressing TWIST or SNAI2. The EMT-linked transcription factors TWIST and SNAI2 were overexpressed in epithelial MCF10A cells. EMT was manifested by acquisition of mesenchymal traits. **a** Fluorescence microscopy for detection of vimentin and E-cadherin, and cell morphology (using phalloidin to stain F-actin), in the parental cells (MCF10A/Par), and cells overexpressing TWIST (MCF10A/TWIST) and SNAI2 (MCF10A/SNAI2). **b** Images (phase-contrast microscopy) showing spheroid formation capacity. **c** Total cellular RNA versus DNA content (Hoechst 33258) in MCF10A/TWIST, compared to MCF10A/Par (flow cytometry). **d** Protein expression of subunit SDHB and SDHC in MCF10A/Par and MCF10A/TWIST cells. **e** Mitochondrial respiration after overexpression of EMT-linked transcription factors. Oxygen consumption rate (OCR) was measured after sequential additions of oligomycin (Oligo), CCCP, rotenone (Rot), and antimycin A (AMA), in DMEM medium. **f** Extracted data from the experiment in (**e**), showing rates of basal and leak (with oligomycin) respiration and respiratory capacity (uncoupled, with CCCP), in the MCF10A/TWIST and MCF10A/SNAI2 cells relative to parental cells (CTR). **g** Leak respiration (with oligomycin) as the percentage of respiratory capacity (uncoupled, with CCCP), from the experiment in (**e**). **h** SDH activity measured in restricted medium (MAS) after the supply of rotenone (Rot), succinate (Succ), ADP, and permabilizing agent (PMP). Oligomycin (Oligo) and antimycin A (AMA) were then added to control mitochondrial integrity and background activity. **i** The diagram shows statistical data from the experiment described in (**h**). Data are shown as mean ± SD (column plots) or mean ± SEM (OCR traces). Student’s *t* test was used for statistical analysis. *p < 0.01

Gene expression analysis suggested that there is a regulatory relationship between EMT-related genes and SDH subunits, especially regarding SDHC, in the breast cancer cohorts of this study. To determine if EMT-linked transcription factors could be involved in SDH downregulation, we overexpressed TWIST and SNAI2 in MCF10A cells (MCF10A/TWIST and MCF10A/SNAI2, respectively). Both the modified cell types presented a switch from epithelial to mesenchymal phenotype, as seen by confocal imaging showing characteristic changes in cell morphology, remodeling of the cytoskeleton, increased level of vimentin, and a reduced level of E-cadherin (Fig. 5a). Induction of EMT was further verified by increased expression levels of vimentin, N-cadherin, Axl, PRXX1, and downregulated E-cadherin, as well as reduced cell proliferation (Additional file 2: Figure S3). In a centrifugation-aided spheroid formation experiment, the parental MCF10A cells formed dense spheroid structures, whereas the MCF10A/TWIST and MCF10A/SNAI2 cells formed less compact structures with loosened cell-cell contact, as expected upon EMT (Fig. 5b). Moreover, a congruent reduction in total RNA level was measured in MCF10A/TWIST cells (Fig. 5c), reflecting a higher content of cells in the state of quiescence due to EMT. Following the verification of EMT in the modified cells, we investigated the effects on SDH. Reduced protein expression of both SDHB and SDHC was detected in the MCF10A/TWIST cells (Fig. 5d). Analysis of oxygen consumption demonstrated that the rates of mitochondrial respiration were lower in MCF10A/TWIST and MCF10A/SNAI2 cells, compared to controls (Fig. 5e–g). The lower rates of leak respiration in the overexpressing cells contradict the possibility that the integrity of mitochondrial inner membrane could be compromised, as this would lead to increased leak respiration due to uncoupling effects. Rather, the lower leak respiration may be explained by a general decrease in mitochondrial respiration. Similar to the previous studies, we then measured SDH-linked respiration in permeabilized cells, in the presence of rotenone, succinate, and ADP (Fig. 5h and i). We found that the activity of SDH was significantly reduced in the MCF10A/TWIST cells, compared to control. Also in these cells, mitochondrial integrity remained intact despite the loss of SDH activity, supported by the normal response to oligomycin in the presence of ADP. In summary, these data suggest that initiation of the EMT program leads to attenuation of SDH.

### TWIST overexpression leads to reduced mitochondrial biomass and changed organelle morphology

**Fig. 6:**
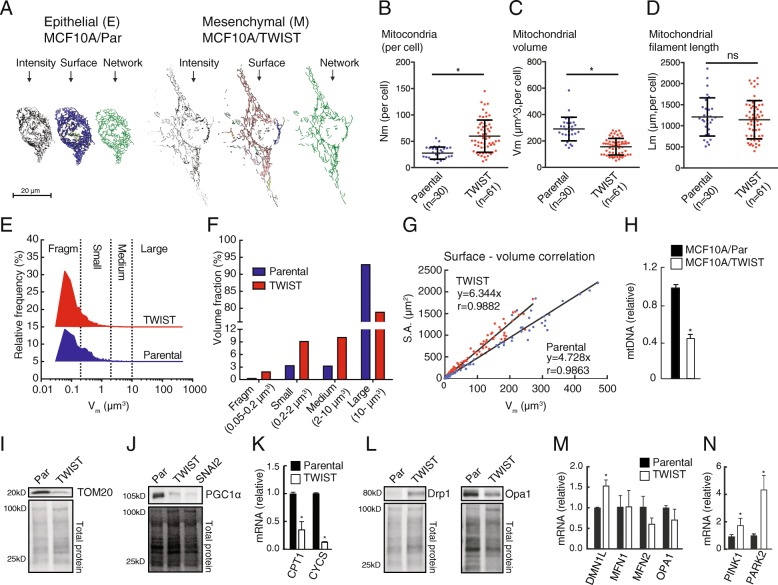
Mitochondrial mass and morphology. Mitochondrial mass and morphology were compared in MCF10A (parental, epithelial) and MFC10A/TWIST (mesenchymal) cells. **a**–**g** Confocal microscopy and quantitative image analysis of immune-stained mitochondria (TOM20 + ATPB). **a** Based on confocal z-stacks, 3D-models of mitochondrial volume and filament structure were generated, as indicated from left to right in the two image panels. **b** Mean number of mitochondria per cell (*N*_*m*_). **c** Mean mitochondrial total volume per cell (*V*_*m*,cell_). **d** Mean mitochondrial total tubule length per cell (*L*_*m*,cell_). **e** Size (volume) frequency distribution comparing mitochondria in MCF10A (parental) versus MCF10A/TWIST cells. **f** Volume fraction analysis of mitochondrial subclasses (size). **g** Surface area (S.A.) to volume (*V*_*m*_) regression analysis of individual mitochondria in MCF10A (parental) and MCF10A/TWIST cells. The analysis comprised (parental/TWIST) 937/3643 mitochondria with total volume 9020/9554 μm^3^, in 30/61 cells (*n*). **h**–**n** Effects of TWIST overexpression on mtDNA and gene expression of mitochondrial proteins. **h** Amount of mtDNA in MCF10A/TWIST relative to parental MCF10A. **i** Protein expression (WB) of TOM20. **j** Protein expression of PGC1α, including MCF10A/SNAI2 cells. **k** mRNA expression of *CPT1* and *CYCS*. **l** Protein expression (WB) of Drp1 (*DMN1L)* and Opa1. **m** mRNA expression of *DMNL1* (Drp1), *OPA1*, *MFN1*, and *MFN2*. **n** mRNA expression of *PINK1* and *PARK2*. Student’s *t* test was used for statistical analysis. Data are shown as mean ± SD. *p < 0.01

Based on the findings suggesting that EMT involves a change in the mitochondrial functional state partly through SDH downregulation, we investigated mitochondrial morphology in MCF10A/TWIST compared to parental MCF10A cells using confocal microscopy and quantitative 3D-image analysis. The mitochondria in MCF10A cells were tubular and formed a compact and continuous reticulum throughout the cytoplasm (Fig. 6a). Mitochondria in MCF10A/TWIST cells were also tubular, but the structures were thinner and there was a fraction of relatively small peripheral organelles dissociated from the major mitochondrial assembly. Quantitative analysis revealed that the number of organelles was significantly higher in MCF10A/TWIST cells, compared to parental cells (Fig. 6b). However, the total mitochondrial volume was smaller (Fig. 6c), though the total mitochondrial tubule length was unchanged (Fig. 6d). Frequency distribution analysis of single mitochondria showed increased proportion of smaller organelles in the MCF10A/TWIST cells (Fig. 6e). Consequently, small- and medium-size mitochondria were found to constitute a larger fraction of the total mitochondrial volume, compared to epithelial MCF10A cells (Fig. 6f). Increased surface-to-volume ratio in MCF10A/TWIST mitochondria further supported a change towards thinner tubular structures compared to the parental phenotype (Fig. 6g). Taken together, these imaging data show that EMT in this model is accompanied by a pronounced decrease in mitochondrial volume and a morphological change from a compact mitochondrial reticulum to a more dispersed and fragmented network of thinner tubules. Congruent with reduced mitochondrial biomass, the MCF10A/TWIST cells had reduced amounts of mitochondrial DNA (mtDNA), TOM20 protein, the transcriptional coactivator and key regulator of mitochondrial biogenesis PGC1α, and mRNA levels of the mitochondrial proteins carnitine palmitoyl transferase 1 (*CPT1A*) and cytochrome *c* (*CYCS*) (Fig. 6h–k). Further supporting the observed changes in mitochondrial dynamics, the mitochondrial fission-related Drp1 protein was found to be upregulated, whereas the mitochondrial fusion protein OPA1 was downregulated (Fig. 6l). Additional mRNA expression analyses confirmed the upregulation of *DMN1L* (DRP1) and indicated a trend for the downregulation of *OPA1* and *MFN2* but not for *MFN1*, in the MCF10A/TWIST cells compared to MCF10A/Par (Fig. 6m). Further, we observed significantly higher *PINK1* and *PARK2* expression in MCF10A/TWIST cells (Fig. 6n). These data supports that EMT involves loss of mitochondrial biomass and more fragmented organelle structure, consistent with reduced respiratory rates.

### Potential links between SDH regulation and EMT activation

**Fig. 7:**
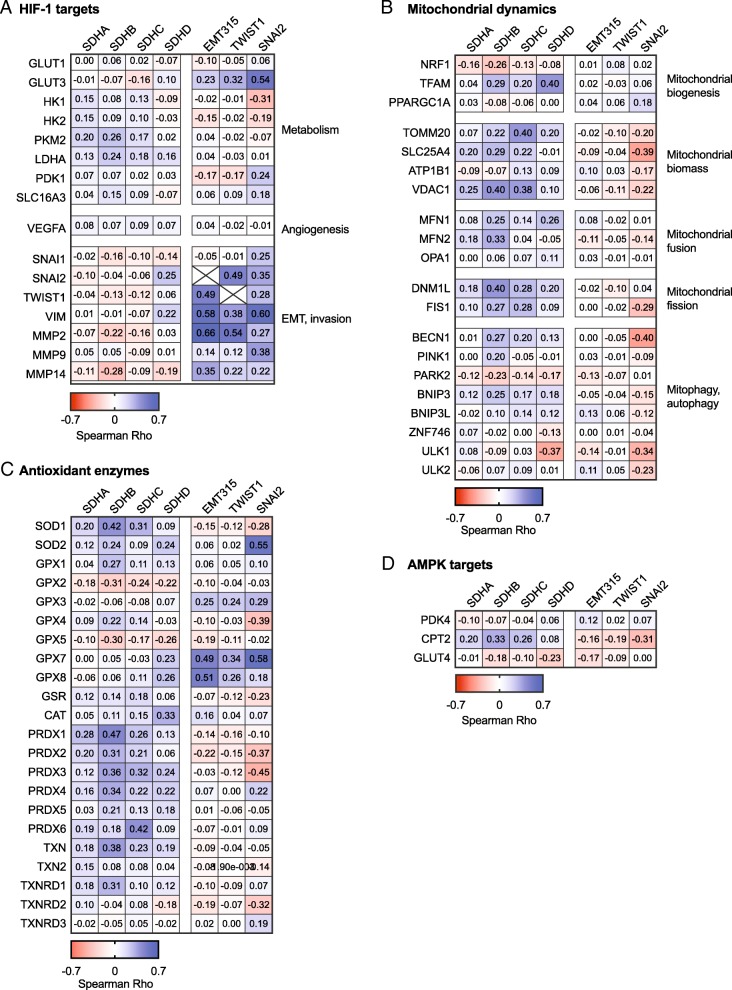
Gene expression analysis of potential links between SDH suppression and EMT activation. Gene expression (mRNA) analysis correlating (Spearman) SDH subunits, EMT signature, TWIST1, and SNAI2 to panels of genes focusing on specific processes, based on data from the Affymetrix breast cancer patient meta cohort (*n* = 3992). The heat map panels reflect the directions and strength (*Rho* value) of the associations. The panels of genes included **a** HIF-1 targets, **b** factors involved in mitochondrial dynamic, **c** antioxidant enzymes, and **d** AMPK targets

Finally, to investigate if the four SDH subunits may have specific impacts on tumor metabolism, mitochondrial dynamics and antioxidant systems related to EMT, we performed correlation analysis including panels of marker genes on the Affymetrix breast cancer cohort. A heat map of the correlation coefficients (Spearman *Rho*) comparing associations between specific genes of interest relative to the SDH subunits and EMT markers is shown in Fig. 7 (the dataset is provided in Additional file 1: Table S2). To clarify, since we aimed to identify potential links between attenuated SDH and EMT activation, we were looking for genes demonstrating an opposite relationship towards SDH compared to EMT (i.e., *Rho* value with opposite signs). In general, *SDHA*, *SDHB*, and *SDHC*, demonstrated similar patterns of association with this panel of genes, in contrast to *SDHD* that showed some divergence compared to the other SDH subunits. This association analysis did not suggest that expression of the EMT315 signature, *TWIST1*, and *SNAI2* was consistently linked to HIF-1 target genes associated with glycolysis, apart from *GLUT3* (Fig. 7a). The expression of *SDHA*, *SDAB*, and *SDHC* tended to be positively associated with the metabolic HIF-1 target genes. Moreover, western blot analysis of HIF-1α revealed no signs of increased protein stabilization in MCF10A overexpressing TWIST, or MCF7 *SDHC/D*^*+/+*^ and MCF7 *SDHD*^*+/−*^ cells (Additional file 2: Figure S4). Hence, although our findings do not directly support that SDH suppression promotes a HIF-1-regulated shift towards increased glycolysis in these tumors, it still remains a possibility. HIF1-regulated genes involved in EMT and invasion demonstrated a clear tendency of inverse regulation relative to *SDHA*, *SDHB*, and *SDHC*, while positively linked to EMT markers. Moreover, the increasing expression of the SDH subunits was associated with increasing expression of markers of mitochondrial biomass (Fig. 7b). These markers generally showed weak inverse relationships with EMT-linked gene expression. SDH subunit expression was also associated with genes involved in both mitochondrial fission and fusion, probably reflecting a parallel regulation of genes encoding mitochondrial proteins. Further, a tendency of positive association with autophagy genes such as *BECN1* and *BNIP3* suggests that SDH downregulation is related to modulation of autophagy, and an inverse association with *PARK2* may suggest that mitochondrial quality control is activated under such conditions. Downregulation of SDH subunits tended to be associated with downregulation of antioxidant systems (i.e., positive associations), apart from *GPX2* and *GPX5* showing inverse relationships with all SDH subunits (Fig. 7c). The effects of such changes may be complex, and these data do not reveal if potential changes in tumor redox state due to reduced SDH subunit expression may be associated with EMT. Although some antioxidant enzymes appeared to linked to EMT, such as *GPX7* and *GPX8*, we did not find clear reciprocal relationships with reduced expression of *SDHC* or the other SDH subunits. Finally, analysis of three anticipated target genes did not indicate that AMPK was coherently activated in the context of SDH suppression but rather showed that these genes were individually regulated, with *CPT2* showing a clear positive relationship with expression of *SDHA*, *SDHB*, and *SDHC* (Fig. 7d).

## Discussion

This study support that attenuation of SDH represents an inherent element and driver mechanism of the EMT program and especially points to SDHC as a contributing factor in the context of breast cancer. Through comprehensive cellular analyses, we characterized regulatory and functional aspects of the relationship between EMT and SDH, and further found the EMT program to involve distinct changes in mitochondrial function and morphology.

Based on previous reports suggesting that mitochondrial dysfunction and *SDHB* mutations promote EMT [4, 37], we hypothesized that altered enzyme function of SDH may be a determining factor and possibly an integral part of EMT in human tumors, which could be linked to an overarching shift in mitochondrial function and dynamics. Thorough analyses in breast cancer patient cohorts revealed a relatively consistent inverse association between EMT and reduced expression of the *SDHC* subunit. Upon molecular and histological classification of the breast cancer cohorts, we found that the relationship between *SDHC* and EMT was stronger in the basal-like subgroup compared to the non-basal-like subgroup. Interestingly, when looking at overall survival, low *SDHC* level was associated with a worse outcome in basal-like breast carcinoma. This finding can imply that suppressed activity of SDH-linked mitochondrial pathways is associated with a worse prognosis in basal-like breast carcinomas. As mitochondrial function is challenging to assess in frozen and paraffin-embedded samples, there is an urgent need for indicative markers reflecting mitochondrial abnormalities. Our results suggest that *SDHC* expression could serve as a potential prognostic marker to enable further discrimination in the basal-like breast carcinoma subgroup. Furthermore, upon histological classification of the *n* = 204 breast cancer cohort, we found the inverse association between SDHC and EMT to be more pronounced in the ductal subgroup, compared to the lobular, but we did not see an impact on the overall survival.

The relationship between SDHC and EMT was supported in subsequent studies revealing that EMT is induced by CRISPR/Cas9-mediated knockdown of *SDHC*, or SDH enzyme inhibition with malonate in respective cell models. Under these conditions, EMT clearly involved inhibition of SDH enzyme activity, as the ability to utilize succinate as respiratory fuel was significantly reduced. These findings extend the potential impact of SDH attenuation on malignant mechanisms beyond the role as tumor suppressor, as they suggest that transcriptional suppression, not only genetic SDH defects, may promote EMT.

Overexpression of EMT-linked transcription factors (TWIST and SNAI2) caused SDH suppression, reduced mitochondrial respiration, decreased amount of mitochondrial biomass (mtDNA and organelle volume), downregulated mitochondrial biogenesis (including PGC1α), and altered organelle structure. Noteworthy, context-dependent changes of mitochondrial functional state are also often accompanied by accordant effects on organelle structure and amount (i.e., mitochondrial dynamics) [38]. Our findings provide mechanistic insights for previous reports elucidating context-dependent links between SDH inhibition, mitochondrial dysfunction, and EMT [4, 13, 22, 39]. The results are also in agreement with findings suggesting that SNAI1 modulates cell metabolism by decreasing respiratory activity via reduced SDH activity [40]. The EMT-linked mechanisms involving reduced SDH activity may share similarities with the action of other mitochondrial tumor suppressors, such as fumarate dehydrogenase, fumarate hydratase, and isocitrate dehydrogenase, as well as OXPHOS defects that contribute to tumor development [18, 41, 42]. According to our findings, it can be speculated that an increased population of smaller mitochondrial organelles may facilitate cellular plasticity accompanying EMT and that this is associated with processes of mitochondrial quality control, as suggested by increased expression of *PINK1* and *PARK2*. This aspect is emphasized by findings in stem cells where mitochondrial quality is supported through asymmetric organelle sorting during cell division [43] and the role of mitochondrial dynamics during embryogenesis [44].

Interestingly, we found a clear relationship between the cells’ ability to form spheroids, wound healing capacity, and EMT state, related to downregulation of *SDHC*. This observation constitutes a convincing example of how metabolic rewiring may represent an integral part of cellular plasticity. It may also incorporate established paradigms of tumor metabolism where suppressed mitochondrial energetics supports biomolecule synthesis or antioxidant defense [45]. Moreover, decreased proliferation and reduced dependency on mitochondrial respiration may constitute a protective adaptation mediating tolerance for a harsh microenvironment, including hypoxia, nutrient deprivation, and therapeutic pressures [46]. This may, however, also open for targeted therapeutic strategies. For instance, RCC tumors with *SDHB* mutations has been shown to be highly dependent on an increased influx of glucose, and glucose interfering drugs have already been proposed as possible therapeutic strategies [47]. Also, it has been shown that mutations in SDH establishes a hypermethylated phenotype in PGL/PCC, possible through the accumulation of succinate [48]. Interestingly, *KRT19*, one of the genes showing the strongest evidence for epigenetic silencing in hypermethylated PGL/PCCs, is closely associated with EMT. This may explain the particularly invasive phenotype of *SDHB*-related tumors.

Gene expression analysis on the Affymetrix breast cancer cohort confirmed that downregulation of *SDHA*, *SDHB*, *SDHC*, and/or *SDHD* is associated with increased expression of EMT and invasion/migration markers and pointed to specific genes that may link to accompanying effects on energy metabolism, mitochondrial quality control, and antioxidant systems to the induction of EMT (Fig. 7). Although HIF-1 and AMPK may be involved in these processes, it appears likely that some of their anticipated target genes may also be regulated by other mechanisms. Moreover, interactions of SDH and other components of the respiratory chain may affect EMT through effects on reactive oxygen species (ROS) generation and scavenging. Such mechanisms likely depend on cellular origin and phenotype, and probably the state of EMT. Our investigations primarily focused on long-term effects associated with EMT. Whether ROS play a role in the induction of EMT, or transition between different states of EMT, would be an interesting aspect for further studies, provided the metabolic links revealed in the presented work.

As EMT is recognized as a driver mechanism of metastasis, therapy resistance, and immune evasion, our findings provide mechanistic support for further investigations of mitochondrial features as potential therapeutic targets. To this end, mitochondria-targeted drugs such as resveratrol and metformin have been reported to inhibit EMT [49], and metformin has a preventive effect on cancer incidence [50]. The presented findings pointing to SDH suppression as an integral part of the EMT process may therefore open for new strategies to prevent and treat malignancies.

## Additional files

Additional file 1: Supplemental methods (PDF 26 kb)

Additional file 2: **Figure S1.** Gene expression correlation analysis extended. **Figure S2.** DNA sequence verification of *SDHC* and *SDHD* CRIPSR/Cas9 modifications. **Figure S3.** Characterization of EMT in MCF10A cells overexpressing TWIST or SNAI2. **Figure S4.** Western blot analysis of HIF-1α in MCF7 and MCF10A cells. **Table S1.** Gene expression correlation analysis, in cell lines. **Table S2.** Gene expression correlation analysis towards specific gene panels. Table S3. Lists of probes, antibodies, and dyes. (PDF 7335 kb)

## Abbreviations

EMT: Epithelial to mesenchymal transition
ER: Estrogen receptor
FH: Fumarate hydratase
GEA: Gene expression analysis
GIST: Gastrointestinal stromal tumor
IDH1: Isocitrate dehydrogenase 1
mtDNA: Mitochondrial DNA
OCR: Oxygen consumption rate
PGL/PCC: Paraganglioma-pheochromocytoma syndrome
qPCR: Quantitative polymerase chain reaction
RCC: Renal cell carcinomas
SDH: Succinate dehydrogenase
TCA cycle: Tricarboxylic acid cycle
TCGA: The cancer genome atlas (https://cancergenome.nih.gov/)
