# Supplementary material for: Epithelial to mesenchymal transition (EMT) is associated with attenuation of succinate dehydrogenase (SDH) in breast cancer through reduced expression of SDHC
Source: Cancer Metab. 2019 Jun 1;7:6. doi: 10.1186/s40170-019-0197-8 (PMC6544948; doi:10.1186/s40170-019-0197-8)
Supplement: Supplementary file 1 — Supplemental methods (PDF 26 kb) [file 40170_2019_197_MOESM1_ESM.pdf]

SUPPLEMENTAL METHODS

Gene expression analysis (GEA) of human breast cancer samples

Breast cancer samples were from a HUS-cohort of 204 patients diagnosed with locally advanced disease and randomized to neoadjuvant epirubicin or paclitaxel monotherapy in a prospective study aiming at identifying predictive factors (termed the n=204 cohort). These patients have been described in detail previously [1]. The breast cancer meta-cohort (*n*=3992) [2] was previously curated from 26 Affymetrix U133A or U133 Plus2 gene expression cohorts publicly available on GEO. Briefly, the 26 cohorts were RMA-normalized, and subsequently standardized using ComBat [3]. An EMT signature score (here called EMT8 score) was calculated as previously reported, including the EMT related genes *CDH1, CTNNA2, CTNNB1, CDH2* and *CDH3* [4, 5] in addition to *TWIST*, *SNAI2* and *KRT19* expression. The sum of the downregulated genes (*CDH1*, *CTNNB1*, *CTNNA2* and *KRT19*) were subtracted from the sum of the upregulated genes (*CDH2, CDH3, SNAI2* and *TWIST*). A second score (here called EMT315 score) is based on the two-sample Kolmogorov-Smirnov test, and estimates the difference in cumulative distribution between consensus epithelial and mesenchymal genes (315 genes in total) derived from 6 carcinoma types [2]. For both EMT signature scores the expression values were mean normalized and scaled to the same standard deviation (SD). Analysis was performed using the statistical software SPSS (Statistical Package of Social Science) version 22.0. Spearman correlation coefficient analyses were applied to assess correlation between SDHs gene expression and EMT315 score. The breast cancer subtypes were classified based on the molecular subgroups published by Perou CM, et al in 2000 [6]. When relevant, the tumors were analyzed as estrogen receptor negative *vs* positive (ER-/+), or as basal like *vs* non-basal like. Additional histological subclassification into ductal and luminal carcinoma was available for the n=204 cohort.

Cell culture

The breast epithelial cells MCF10A (ATCC, Manassas, VA) were grown in DMEM/F12 (Dulbecco's Modified Eagle's Medium/Nutrient F-12 Ham, Sigma) containing 50 µg/mL penicillin/streptomycin (P-0781, Sigma), 5 % horse serum (B15021, PAA/GE Healthcare, Little Chalfont, UK), 20 ng/ml EGF (E9644, Sigma), 10 µg/ml insulin (I1882, Sigma), 0.5 µg/ml hydrocortisone (H0888, Sigma) and 100 ng/ml cholera toxin (C8052, Sigma). The MCF7 breast cancer cell line (ATCC) was cultured in EMEM (BL12-125F, ATCC) which was supplemented with 10 % heat-inactivated fetal bovine serum (FBS, SH30079.03, GE Healthcare Hyclone), 1 % L-glutamine (G7513, Sigma) and 50 µg/mL penicillin/streptomycin. All cells were routinely incubated in 5 % CO_2 at 37ºC. Cells subjected to gene editing by CRISPR/Cas9 were STR-profiled before and after editing. The GlobalFiler™ PCR Amplification Kit and GeneScan 600 LIZ dye size standard (Life technologies), was used according to the manufacturer's instructions. Results were assessed by the GeneMapper®ID-X software (v.1.4) and observed alleles were compared to the theoretical alleles available at (www.atcc.org). The experiments were performed with cultures passaged less than 10 times from thawing.

Mitochondrial respiration analysis

Oxygen consumption rate (OCR) was measured using the Seahorse XFe96 Analyzer (Agilent, Santa Clara, CA), according to the manufacturers protocols and previous description [7, 8]. All materials were from Sigma-Aldrich (St. Louis, MO) unless otherwise stated. Cell number and concentrations of compounds were optimized for each cell type. The cells were transferred to the analysis plate the day before measurement, and incubated overnight. For analysis of SDH-linked activity, the cells were permeabilized to facilitate cellular uptake of succinate and ADP, by adding the Seahorse XF plasma membrane permeabilizer (PMP) (Agilent; MCF10A, 1.5 nM; MCF7, 1 nM) immediately before or under the analysis, as indicated. The cell plate was

transferred to the instrument after replacing the culture medium with the mitochondrial assay solution (MAS), containing mannitol (229 mM), sucrose (70 mM), KH_2PO_4 (10 mM), $MgCl_2$ (5 mM), HEPES (2 mM), EGTA (1 mM) and 0.2 % fatty acid free BSA. Succinate (10 mM), ADP (4 mM), oligomycin (3 µM), antimycin A (10 µM) and rotenone (10 µM) were added as indicated in the figures. For studies of mitochondrial respiration in intact cells, OCR was recorded after successive administrations of oligomycin (3 µM), CCCP (MCF10A, 1.5 µM; MCF7, 0.75µM), rotenone (1 µM) and antimycin A (1 µM). These studies were performed in cell culture medium (D5030) supplemented with glucose (10 mM), pyruvate (2 mM) and glutamine (4 mM) as the major substrates; or in MAS medium without BSA, supplemented with pyruvate (2 mM), glutamine (4 mM) or succinate (10 mM). All data were normalized to cell number using Hoechst 33342 (Thermo Fisher Scientific), or protein content, measured using the Pierce® BCA Protein Assay Kit (Thermo Fisher Scientific, Waltham, MA).

Confocal microscopy and quantitative image analysis of mitochondria

Cells were plated at a density of 50000 cells/well on coverslips placed in 24-well cell culture plates. Following over night incubation, the cells were fixed in 4 % PFA for 15 min and permeabilized with 0.5 % Triton X-100 for 4 min. After washing and blocking in TBS-T/5%BSA the cells were incubated with a mixture of primary antibodies (1:100 TOM20, FL145 Santa Cruz Biotech, Dallas, Texas; 1:500 ATPB, AB5452 Abcam, Cambridge, UK) in the blocking solution for 1 h at room temperature, and then over night at 4 ^{o}C. After washing and blocking, the cells were incubated with secondary antibodies (Alexa 546 anti-rabbit and Alexa 647 anti-mouse, (Molecular Probes, Eugene, OR) for 1 h at room temperature. The coverslips were mounted in Prolong Diamond antifade with DAPI (Thermo Fisher Scientific, Waltham, MA).

Confocal z-stacks were acquired on a confocal Leica TCS S5 microscope (Leica microsystems, Wetzlar, Germany), using a Lambda 63x 1.4 NA oil objective. Image pixel size

was 0.0944 µm (x and y) and bit depth 12, z-spacing 0.34 µm. Image processing and quantitative analysis were performed using the Image-Pro Plus software (version 7.0) (Media Cybernetics, Inc., Washington, USA), as described previously [9]. The acquired 12 bit z-stacks were background corrected (fixed level within each experiment) and processed by 3D blind deconvolution. Single cells were manually segmented to enable analysis of individual cells. The single cell z-stacks were loaded into the 3D module of the software and analyzed. The surface level was fixed for the experiments in this study. Structures larger than 0.05 μm^3 were accounted as mitochondrial objects. Mitochondrial network analysis was performed as previously described [7, 9].

REFERENCES

1. Chrisanthar R, Knappskog S, Løkkevik E, Anker G, Østenstad B, Lundgren S, Risberg T, Mjaaland I, Skjønsberg G, Aas T, et al: **Predictive and Prognostic Impact of TP53 Mutations and MDM2 Promoter Genotype in Primary Breast Cancer Patients Treated with Epirubicin or Paclitaxel.** *PLoS ONE* 2011, **6:**e19249.
2. Tan TZ, Miow QH, Miki Y, Noda T, Mori S, Huang RY-J, Thiery JP: **Epithelial-mesenchymal transition spectrum quantification and its efficacy in deciphering survival and drug responses of cancer patients.** *EMBO Molecular Medicine* 2014, **6:**1279-1293.
3. Johnson WE, Li C, Rabinovic A: **Adjusting batch effects in microarray expression data using empirical Bayes methods.** *Biostatistics* 2007, **8:**118-127.
4. Wik E, Raeder MB, Krakstad C, Trovik J, Birkeland E, Hoivik EA, Mjos S, Werner HM, Mannelqvist M, Stefansson IM, et al: **Lack of estrogen receptor-alpha is associated with epithelial-mesenchymal transition and PI3K alterations in endometrial carcinoma.** *Clin Cancer Res* 2013, **19:**1094-1105.
5. Salvesen HB, Carter SL, Mannelqvist M, Dutt A, Getz G, Stefansson IM, Raeder MB, Sos ML, Engelsen IB, Trovik J, et al: **Integrated genomic profiling of endometrial carcinoma associates aggressive tumors with indicators of PI3 kinase activation.** *Proc Natl Acad Sci U S A* 2009, **106:**4834-4839.
6. Perou CM, Sørlie T, Eisen MB, van de Rijn M, Jeffrey SS, Rees CA, Pollack JR, Ross DT, Johnsen H, Akslen LA, et al: **Molecular portraits of human breast tumours.** *Nature* 2000, **406:**747.
7. Hodneland Nilsson LI, Nitschke Pettersen IK, Nikolaisen J, Micklem D, Avsnes Dale H, Vatne Røsland G, Lorens J, Tronstad KJ: **A new live-cell reporter strategy to simultaneously monitor mitochondrial biogenesis and morphology.** *Scientific Reports* 2015, **5:**17217.
8. VanLinden MR, Dölle C, Pettersen IKN, Kulikova VA, Niere M, Agrimi G, Dyrstad SE, Palmieri F, Nikiforov AA, Tronstad KJ, Ziegler M: **Subcellular Distribution of NAD+ between Cytosol and Mitochondria Determines the Metabolic Profile of Human Cells.** *Journal of Biological Chemistry* 2015, **290:**27644-27659.

9. Nikolaisen J, Nilsson LIH, Pettersen IKN, Willems PHGM, Lorens JB, Koopman WJH, Tronstad KJ: **Automated Quantification and Integrative Analysis of 2D and 3D Mitochondrial Shape and Network Properties.** *PLoS ONE* 2014, **9:**e101365.
