# Supplementary material for: Epithelial to mesenchymal transition (EMT) is associated with attenuation of succinate dehydrogenase (SDH) in breast cancer through reduced expression of SDHC
Source: Cancer Metab. 2019 Jun 1;7:6. doi: 10.1186/s40170-019-0197-8 (PMC6544948; doi:10.1186/s40170-019-0197-8)
Supplement: Supplementary file 2 — Figure S1. Gene expression correlation analysis extended. Figure S2. DNA sequence verification of SDHC and SDHD CRIPSR/Cas9 modifications. Figure S3. Characterization of EMT in MCF10A cells overexpressing TWIST or SNAI2. Figure S4. Western blot analysis of HIF-1α in MCF7 and MCF10A cells. Table S1. Gene expression correlation analysis, in cell lines. Table S2. Gene expression correlation analysis towards specific gene panels. Table S3. Lists of probes, antibodies, and dyes. (PDF 7335 kb) [file 40170_2019_197_MOESM2_ESM.pdf]

Figure S1

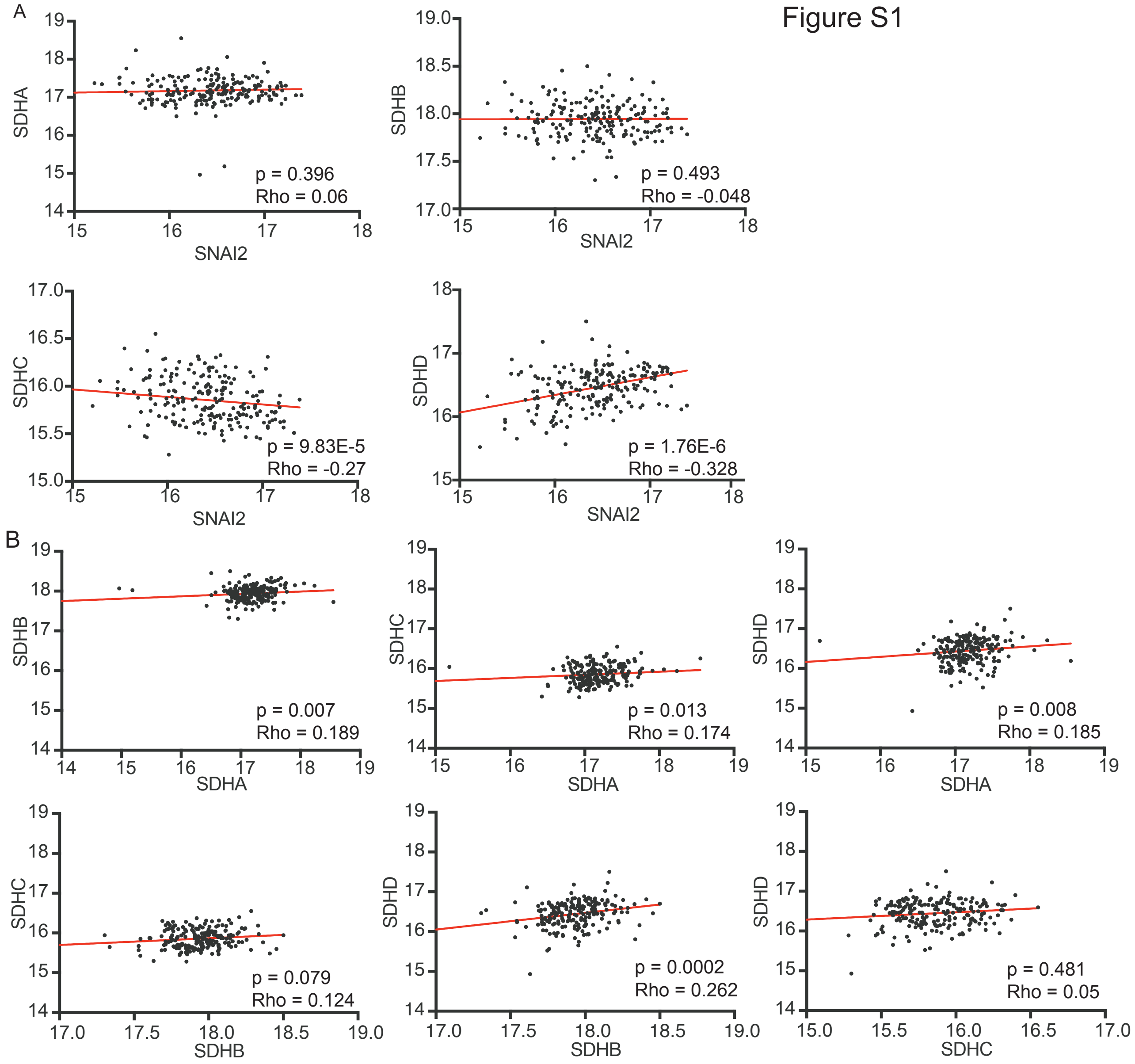

Figure S1: (A) Correlation analysis (Spearman) between the expression of the four SDH subunits relative to SNAI2 in the n=204 breast cancer cohort. (B) Correlation analysis (Spearman) between the individual SDH subunits in the n=204 breast cancer cohort.

Figure S2

A

Homo sapiens chromosome 1, GRCh38.p7 Primary Assembly

Sequence ID: NC_000001.11 Length: 248956422 Number of Matches: 1

Range 1: 161328241 to 161328620 GenBank Graphics ▼ Next Match ▲ Previous Match

Score	Expect	Identities	Gaps	Strand
614 bits(332)	8e-173	366/380(96%)	11/380(2%)	Plus/Plus

Features: succinate dehydrogenase cytochrome b560 subunit, mitochon...
succinate dehydrogenase cytochrome b560 subunit, mitochon...

```
Query  546        attcgcccttctcggcctcccaaagagctgagattacaggcctgagcaaccatgcctggc  605
                  || |||||  ||||||||||||||||||||||||||||||||||||||||||||||||||
Sbjct  161328241  ATCCGCCCGCCTCGGCCTCCCAAAGAGCTGAGATTACAGGCCTGAGCAACCATGCCTGGC  161328300

Query  606        TTGGTATTGCAAAATATTGACTTAATAAAACGTTATGCAAAATATTAAACCAAGTTTACT  665
                  ||||||||||||||||||||||||||||||||||||||||||||||||||||||||||||
Sbjct  161328301  TTGGTATTGCAAAATATTGACTTAATAAAACGTTATGCAAAATATTAAACCAAGTTTACT  161328360

Query  666        TTTAGTTATTTTCAAACGGTCTGGTTTTATTTTAGTGCTGTTCCTTTGGGAACCACGGCC  725
                  ||||||||||||||||||||||||||||||||||||||||||||||||||||||||||||
Sbjct  161328361  TTTAGTTATTTTCAAACGGTCTGGTTTTATTTTAGTGCTGTTCCTTTGGGAACCACGGCC  161328420

Query  726        AAAGAAGAGATGGAGCGGTTCTGGAATAAGAATATAGGTTCAAACCGTCCTCT-------  778
                  |||||||||||||||||||||||||||||||||||||||||||||||||||||
Sbjct  161328421  AAAGAAGAGATGGAGCGGTTCTGGAATAAGAATATAGGTTCAAACCGTCCTCTGTCTCCC  161328480

Query  779        ----TTACTATCTACAGGTAAGGAAGGATTCTGGAGCCAGAGAATCTAGAGGTAGTGGGT  834
                      ||||||||||||||||||||||||||||||||||||||||||||||||||||||||
Sbjct  161328481  CACATTACTATCTACAGGTAAGGAAGGATTCTGGAGCCAGAGAATCTAGAGGTAGTGGGT  161328540

Query  835        GAAAGTTCTGAAGGTTGATCTTTAGCCTACTTGATACTTCCCTCACTTTTACTCAACCAA  894
                  ||||||||||||||||||||||||||||||||||||||||||||||||||||||||||||
Sbjct  161328541  GAAAGTTCTGAAGGTTGATCTTTAGCCTACTTGATACTTCCCTCACTTTTACTCAACCAA  161328600

Query  895        AATACTGCTATGTAGATGAG  914
                  ||||||||||||||||||||
Sbjct  161328601  AATACTGCTATGTAGATGAG  161328620
```

B

Homo sapiens chromosome 11, alternate assembly CHM1_1.1

Sequence ID: NC_018922.2 Length: 134889443 Number of Matches: 1

Range 1: 111840691 to 111841012 GenBank Graphics ▼ Next Match ▲ Previous Match

Score	Expect	Identities	Gaps	Strand
468 bits(518)	5e-129	292/322(91%)	29/322(9%)	Plus/Plus

Features: succinate dehydrogenase [ubiquinone] cytochrome b small s...
succinate dehydrogenase [ubiquinone] cytochrome b small s...

```
Query  64         CTCTCGACTTCCGGTTCACCCAGCATTTCCACTTCCCTGTTTTCTTTCGTCGTCGTGGGT  123
                  |||||||||||||||||||||||||||||| |||||||||||||||||||||||||||||
Sbjct  111840691  CTCTCGACTTCCGGTTCACCCAGCATTTCCTCTTCCCTGTTTTCTTTCGTCGTCGTGGGT  111840750

Query  124        GGGAATTGTCGCCTAAGTGGTTCCGGGTTGGTGGATGACCTTGAGCCCTCAGGAACGAGA  183
                  ||||||||||||||||||||||||||||||||||||||||||||||||||||||||||||
Sbjct  111840751  GGGAATTGTCGCCTAAGTGGTTCCGGGTTGGTGGATGACCTTGAGCCCTCAGGAACGAGA  111840810

Query  184        TGGCGGTTCTCTGGAGGCTGAGTGCCGTTTGCGGTGCCCTAG------------------  225
                  ||||||||||||||||||||||||||||||||||||||||||
Sbjct  111840811  TGGCGGTTCTCTGGAGGCTGAGTGCCGTTTGCGGTGCCCTAGGAGGCCGAGGTGAGGGGT  111840870

Query  226        -----------GAGGTGCTTAGCGTAGCCTCCAGCCAGGGAAGGGGATGGAAGTGAGGAC  274
                             |||||||||||||||||||||||||||||||||||||||||||||||||
Sbjct  111840871  CTTCCCACCCTGAGGTGCTTAGCGTAGCCTCCAGCCAGGGAAGGGGATGGAAGTGAGGAC  111840930

Query  275        TCATCTGCCGGGTGGGAGATCTCTTGAGGAGAAGAAAATACCGAAATCACAGCAATGACC  334
                  ||||||||||||||||||||||||||||||||||||||||||||||||||||||||||||
Sbjct  111840931  TCATCTGCCGGGTGGGAGATCTCTTGAGGAGAAGAAAATACCGAAATCACAGCAATGACC  111840990

Query  335        ACTGTAGTCTAGGGGTCCAGAT  356
                  ||||||||||||||||||||||
Sbjct  111840991  ACTGTAGTCTAGGGGTCCAGAT  111841012
```

C

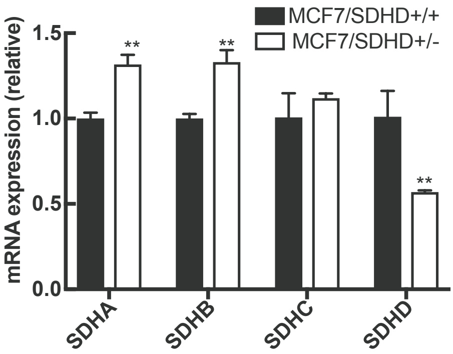

Figure S2: (A) Sequence alignment of the SDHC gene in CRIPSR/Cas9- modified MCF7 cells and the SDHC wild-type gene, revealing an 11bp frameshift deletion. (B)Sequence alignment of the SDHD gene in CRIPSR/Cas9-modified MCF7 cells and the SDHD wild-type gene, revealing an 29bp frameshift deletion. (C) qPCR analysis of SDHA-D in MCF7 SDHD +/- cells.

Figure S3

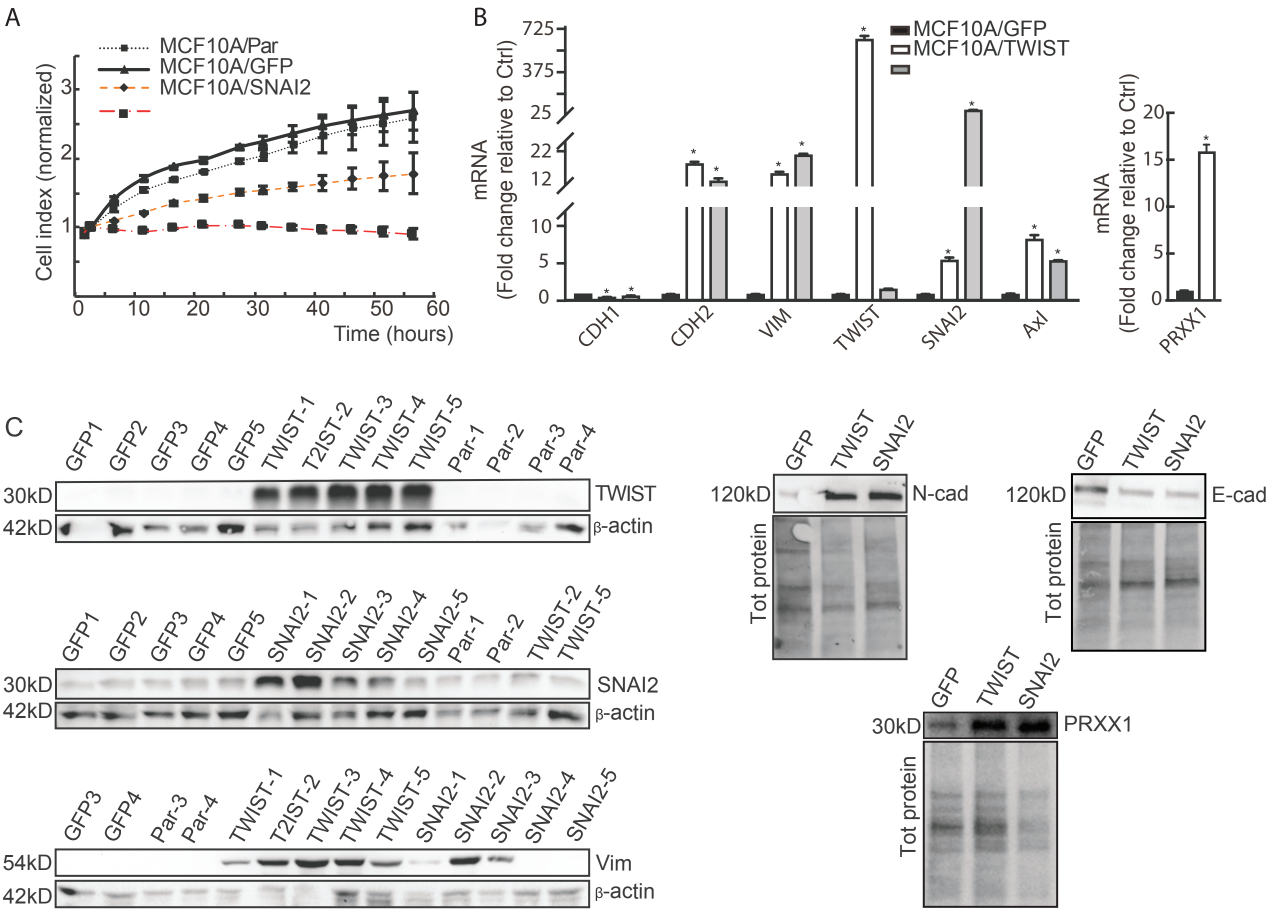

Figure S3: Characterization of the EMT process in MCF10A cells upon overexpression of TWIST and SNAI2 (Slug). (A) Cell proliferation was measured by the use of the xCelligence RTCA DP instrument (ACEA Bioscience, San Diego, CA), which measures cellular impedance (expressed as “Cell index”). The cells (MCF10A, MCF10A/GFP, MCF10A/TWIST, MCF10A/SNAI2) were seeded at a density of 10 000 cells/well. (B) mRNA and (C) protein expression of TWIST and SNAI2, and epithelial (E-cadherin (CDH1)) and mesenchymal (N-cadherin (CDH2), Vimentin (VIM), Twist, SNAI2, Axl and PRXX1) markers. The numbers at the end of the sample name in the western blots refer to passage number after transduction.

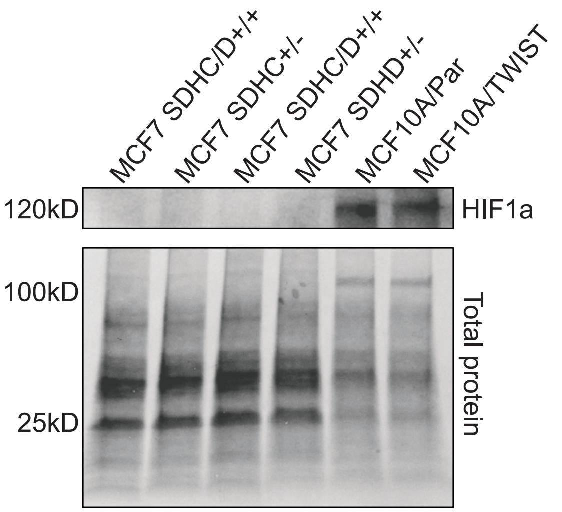

Figure S4: Western blot analysis of HIF1α in MCF7 SDHC/D+/+, MCF7 SDHC+/-, and MCF7 SDHD+/- cells, and in MCF10A/Par and MCF10A/TWIST cells.

Table S1

A

Category	SDHAvsEMT	SDHAvsEMT	SDHBvsEMT	SDHBvsEMT	SDHCvsEMT	SDHCvsEMT	SDHDvsEMT	SDHDvsEMT
	Spear.Rho	Spear.pv	Spear.Rho	Spear.pv	Spear.Rho	Spear.pv	Spear.Rho	Spear.pv
Breast cancer cell	´-0.1578	0.2686	0.0957	0.504	´-0.5174	0.0001	-0.0353	0.8060

B

Disease	SDHAvsEMT	SDHAvsEMT	SDHBvsEMT	SDHBvsEMT	SDHCvsEMT	SDHCvsEMT	SDHDvsEMT	SDHDvsEMT
	Spear.Rho	Spear.pv	Spear.Rho	Spear.pv	Spear.Rho	Spear.pv	Spear.Rho	Spear.pv
Breast invasive carcinoma (BRCA)	´-0.0777	0.0068	´-0.0611	0.0335	´-0.3373	1.2E-33	0.3676	4.51E-40

Table S1: Spearman correlation analysis was applied to assess associations between SDH subunit gene expression and EMT315 score on (A) Encyclopedia breast cancer cell line collection (56 cell lines) and for (B)TCGA RNA-seq extracted from GDAC version 2018_01_28 from breast cancer carcinoma cohort (7).

Table S2

	SDHA	SDHA	SDHB	SDHB	SDHC	SDHC	SDHD	SDHD	SNAI2	SNAI2	TWIST1	TWIST1	EMT	EMT
**Gene**	Rho	pv	Rho	pv	Rho	pv	Rho	pv	Rho	pv	Rho	pv	Rho	pv
SLC2A1	0,002	9,0E-01	0,063	6,7E-05	0,023	1,43E-01	-0,068	1,70E-05	-0,0953	1,45E-09	-0,048	2,22E-03	0,060	1,31E-04
SLC2A3 (GI	-0,012	4,4E-01	-0,070	8,8E-06	-0,160	1,84E-24	0,102	9,68E-11	0,2313	6,79E-50	0,324	1,28E-98	0,540	3,5E-302
HK1	0,151	6,5E-22	0,084	1,1E-07	0,127	6,01E-16	-0,091	7,16E-09	-0,0236	1,35E-01	-0,013	0,423948	-0,308	4,59E-89
HK2	0,147	6,4E-21	0,087	4,0E-08	0,098	5,17E-10	-0,031	4,80E-02	-0,1503	1,01E-21	-0,024	0,125805	-0,192	9,43E-35
PKM2	0,203	1,2E-38	0,260	4,0E-63	0,166	3,69E-26	0,018	2,46E-01	0,0434	0,00596	-0,020	2,13E-01	-0,066	2,86E-05
LDHA	0,126	1,2E-15	0,237	1,7E-52	0,181	7,85E-31	0,162	4,05E-25	0,0413	8,83E-03	-0,029	6,61E-02	0,008	6,14E-01
PDK1	0,069	1,1E-05	0,071	7,6E-06	0,020	1,95E-01	0,027	0,084600	-0,1724	3,87E-28	-0,170	2,00E-27	0,244	1,92E-55
SLC16A3	0,041	9,7E-03	0,145	2,0E-20	0,089	1,56E-08	-0,071	6,08E-06	0,0590	0,00018	0,088	2,74E-08	0,183	1,64E-31
SLC16A4	-0,151	7,5E-22	-0,051	1,2E-03	0,043	6,41E-03	0,151	8,85E-22	0,2441	1,61E-55	0,128	4,64E-16	-0,025	1,13E-01
VEGFA	0,076	1,2E-06	0,067	2,1E-05	0,093	4,00E-09	0,074	3,11E-06	0,0412	0,00910	-0,016	3,26E-01	-0,006	6,83E-01
SNAI1	-0,023	1,4E-01	-0,156	3,6E-23	-0,099	3,23E-10	-0,138	1,71E-18	-0,0459	3,64E-03	-0,005	7,65E-01	0,252	2,84E-59
SNAI2	-0,095	1,6E-09	-0,045	4,7E-03	-0,062	9,56E-05	0,250	2,97E-58	1,0000	0	0,487	8,1E-238	0,351	5,7E-117
TWIST1	-0,043	6,3E-03	-0,126	9,3E-16	-0,123	5,09E-15	0,059	0,000205	0,4867	8,1E-238	1,000	0,0E+00	0,280	3,00E-73
VIM	0,009	5,5E-01	-0,007	6,6E-01	-0,066	2,51E-05	0,215	2,29E-43	0,5766	0	0,377	1,5E-135	0,600	0,0E+00
MMP2	-0,066	3,2E-05	-0,224	6,5E-47	-0,158	7,90E-24	0,030	0,060545	0,6638	0,0E+00	0,540	1,9E-302	0,273	1,14E-69
MMP9	0,045	4,2E-03	0,055	5,2E-04	-0,088	2,01E-08	0,007	6,69E-01	0,1367	3,30E-18	0,117	1,25E-13	0,384	1,8E-141
MMP14	-0,112	1,2E-12	-0,281	6,9E-74	-0,086	5,20E-08	-0,193	4,87E-35	0,3520	2,1E-117	0,217	4,69E-44	0,220	4,71E-45
PDK4	-0,099	2,7E-10	-0,075	2,0E-06	-0,039	1,25E-02	0,056	0,000389	0,1151	2,60E-13	0,019	2,29E-01	0,069	1,19E-05
PPARGC1A	0,027	8,4E-02	-0,083	1,2E-07	-0,058	2,50E-04	0,001	0,943557	0,0402	1,08E-02	0,056	3,60E-04	0,181	9,54E-31
CPT2	0,201	1,0E-37	0,330	2E-102	0,263	2,16E-64	0,082	2,33E-07	-0,1565	2,03E-23	-0,187	5,82E-33	-0,306	8,03E-88
SLC2A4 (GI	-0,013	4,1E-01	-0,183	1,1E-31	-0,105	2,60E-11	-0,234	7,59E-51	-0,1735	1,73E-28	-0,087	3,37E-08	-0,001	9,32E-01
NRF1	-0,164	1,1E-25	-0,260	3,9E-63	-0,130	1,45E-16	-0,083	1,46E-07	0,0070	0,65835	0,078	6,43E-07	0,020	1,97E-01
TFAM	0,037	2,0E-02	0,286	2,7E-76	0,200	1,70E-37	0,398	3,3E-152	0,0176	0,26405	-0,031	0,04850	0,058	2,12E-04
PPARGC1A	0,027	8,4E-02	-0,083	1,2E-07	-0,058	0,00025	0,001	9,44E-01	0,0402	1,08E-02	0,056	0,00036	0,181	9,54E-31
TOMM20	0,071	6,1E-06	0,215	2,8E-43	0,396	9,6E-151	0,195	1,00E-35	-0,0208	0,187073	-0,104	3,44E-11	-0,196	5,17E-36
SLC25A4	0,204	4,7E-39	0,291	5,7E-79	0,220	3,44E-45	-0,011	0,503160	-0,0858	5,14E-08	-0,037	2,04E-02	-0,388	2,2E-144
ATP1B1	-0,087	3,8E-08	-0,074	2,6E-06	0,131	7,50E-17	0,085	5,83E-08	0,0984	4,21E-10	0,033	0,035276	-0,167	1,44E-26
VDAC1	0,253	8,2E-60	0,400	4E-154	0,378	9,4E-137	0,100	2,45E-10	-0,0576	2,59E-04	-0,108	6,18E-12	-0,222	4,70E-46
MFN1	0,085	6,9E-08	0,248	3,9E-57	0,139	9,36E-19	0,261	1,66E-63	0,0828	1,51E-07	-0,020	2,10E-01	0,009	0,560201
MFN2	0,176	2,7E-29	0,327	6E-101	0,043	5,91E-03	-0,047	3,19E-03	-0,1123	9,79E-13	-0,052	0,00096	-0,145	3,07E-20
OPA1	-0,003	8,7E-01	0,060	1,3E-04	0,071	6,84E-06	0,108	5,92E-12	0,0323	4,10E-02	-0,007	6,40E-01	-0,014	0,35938
DNM1L	0,177	1,0E-29	0,400	3E-154	0,283	1,01E-74	0,197	2,79E-36	-0,0233	0,139847	-0,098	5,10E-10	0,039	1,28E-02
FIS1	0,103	5,8E-11	0,268	5,5E-67	0,280	5,60E-73	0,087	3,93E-08	0,0045	0,774329	-0,016	3,12E-01	-0,290	1,03E-78
SOD1	0,199	3,6E-37	0,417	5E-169	0,313	8,70E-92	0,091	6,86E-09	-0,1501	1,17E-21	-0,119	3,09E-14	-0,281	1,29E-73
SOD2	0,123	4,8E-15	0,242	1,9E-54	0,089	1,82E-08	0,241	4,45E-54	0,0572	2,87E-04	0,018	2,64E-01	0,553	0,0E+00
GPX1	0,038	1,7E-02	0,272	2,9E-69	0,108	8,49E-12	0,133	2,85E-17	0,0596	0,000160	0,052	0,00092	0,104	4,10E-11
GPX2	-0,176	3,5E-29	-0,309	2,7E-89	-0,242	1,55E-54	-0,219	7,78E-45	-0,1002	2,00E-10	-0,038	1,64E-02	-0,035	2,75E-02
GPX3	-0,017	2,7E-01	-0,057	2,7E-04	-0,082	2,29E-07	0,073	3,97E-06	0,2459	2,44E-56	0,242	1,25E-54	0,294	8,09E-81
GPX4	0,093	3,8E-09	0,217	8,3E-44	0,139	7,20E-19	-0,033	3,88E-02	-0,0999	2,29E-10	-0,034	0,02971	-0,386	1,0E-142
GPX5	-0,100	2,4E-10	-0,303	2,8E-86	-0,172	5,36E-28	-0,258	4,25E-62	-0,1871	6,11E-33	-0,109	4,77E-12	-0,017	0,277220
GPX7	0,002	9,1E-01	0,049	2,1E-03	-0,026	0,096863	0,232	2,76E-50	0,4882	1,5E-239	0,336	2,6E-106	0,580	0,0E+00
GPX8	-0,060	2,7E-02	0,063	2,1E-02	0,109	6,49E-05	0,262	1,72E-22	0,5077	5,11E-89	0,258	6,23E-22	0,178	4,44E-11
GSR	0,125	2,3E-15	0,138	1,5E-18	0,184	6,16E-32	0,057	0,000328	-0,0721	4,86E-06	-0,123	6,46E-15	-0,230	1,61E-49
CAT	0,048	2,5E-03	0,109	3,9E-12	0,151	7,93E-22	0,326	5,6E-100	0,1630	2,67E-25	0,045	4,66E-03	0,075	2,01E-06
PRDX1	0,277	9,2E-72	0,467	2E-216	0,258	7,05E-62	0,126	1,16E-15	-0,1448	2,93E-20	-0,159	3,39E-24	-0,096	1,21E-09
PRDX2	0,198	9,7E-37	0,313	3,3E-92	0,213	3,26E-42	0,057	0,000297	-0,2172	4,72E-44	-0,148	4,82E-21	-0,366	1,3E-127
PRDX3	0,124	3,9E-15	0,356	4E-120	0,318	7,43E-95	0,244	1,14E-55	-0,0337	0,032918	-0,121	1,85E-14	-0,453	5,0E-202
PRDX4	0,164	1,1E-25	0,342	2E-110	0,218	2,72E-44	0,223	1,55E-46	0,0677	1,76E-05	0,001	9,63E-01	0,224	6,93E-47
PRDX5	0,033	2,3E-01	0,205	2,7E-14	0,125	4,21E-06	0,176	7,37E-11	0,0055	0,840649	-0,060	0,027751	-0,053	0,05155
PRDX6	0,192	1,0E-34	0,183	1,3E-31	0,422	1,1E-172	0,087	3,73E-08	-0,0669	2,23E-05	-0,015	0,353144	0,088	1,96E-08
TXN	0,182	3,7E-31	0,384	2E-141	0,229	5,45E-49	0,192	1,69E-34	-0,0946	1,92E-09	-0,038	1,52E-02	-0,047	2,72E-03
TXN2	0,150	1,4E-21	0,083	1,6E-07	0,085	7,32E-08	0,042	7,36E-03	-0,0806	3,16E-07	-0,002	9,04E-01	-0,143	7,91E-20
TXNRD1	0,176	2,2E-29	0,308	4,6E-89	0,100	2,14E-10	0,124	3,52E-15	-0,1007	1,63E-10	-0,087	3,76E-08	0,071	7,50E-06
TXNRD2	0,104	4,9E-11	-0,040	1,1E-02	0,076	1,30E-06	-0,178	8,42E-30	-0,1949	1,20E-35	-0,075	2,13E-06	-0,324	6,37E-99
TXNRD3	-0,023	1,4E-01	-0,054	6,8E-04	0,049	0,00183	-0,022	1,55E-01	0,0214	1,75E-01	0,003	8,70E-01	0,186	1,09E-32
BECN1	0,008	6,0E-01	0,269	1,0E-67	0,205	2,50E-39	0,129	2,64E-16	-0,0048	0,76329	-0,053	7,50E-04	-0,404	1,9E-157
PINK1	0,001	9,4E-01	0,205	3,3E-39	-0,050	0,001571	-0,011	0,468433	0,0259	0,10085	-0,011	5,00E-01	-0,094	2,82E-09
PARK2	-0,120	2,4E-14	-0,225	3,0E-47	-0,144	4,02E-20	-0,173	1,96E-28	-0,1276	4,88E-16	-0,071	6,20E-06	0,005	7,42E-01
BNIP3	0,125	1,9E-15	0,245	4,4E-56	0,174	9,31E-29	0,178	5,42E-30	-0,0546	5,34E-04	-0,043	6,67E-03	-0,154	7,39E-23
BNIP3L	-0,022	1,6E-01	0,102	1,1E-10	0,142	1,32E-19	0,123	5,36E-15	0,1308	9,02E-17	0,062	7,57E-05	-0,124	3,36E-15
ZNF746	0,075	6,2E-03	-0,017	5,3E-01	-0,003	0,902185	-0,125	4,02E-06	-0,0014	0,959629	0,010	0,72092	-0,040	0,14585
ULK1	0,081	2,9E-07	-0,088	2,8E-08	0,029	7,06E-02	-0,366	9,3E-128	-0,1373	2,40E-18	-0,007	0,63843	-0,341	5,2E-110
ULK2	-0,057	2,8E-04	0,073	3,2E-06	0,091	7,23E-09	0,012	0,456505	0,1150	2,73E-13	0,048	2,36E-03	-0,231	1,28E-49

Table S2: Gene expression (mRNA) correlation analysis (Spearman) between SDH subunits and listed genes in the Affymetrix breast cancer patient meta cohort (n=3,992)

A

Table S3

Probes all from applied biosystems

Gene name	Gene	Catalog number
AXL receptor tyrosine kinase	AXL	hs01064444
Carnitine palmitoyltransferase 1A	CPT1A	hs00912671
Cytochrome c	CYCS	hs01588974
Dynamin 1 like	DNM1L	hs01552605
E-cadherin	CHD1	hs01023894
Eukaryotic 18S rRNA Endogenous Control	18S	4310893E
Mitochondrially encoded NADH dehydrogenase 1	MT-ND1	hs02596873
Mitofusin 1	MFN1	hs00966851
Mitofusin 2	MFN2	hs00208382
N-cadherin	CDH2	hs00983056
OPA1, mitochondrial dynamin like GTPase	OPA1	hs01047018
Parkin RBR E3 ubiquitin protein ligase	PARK2	hs01038325
PTEN induced putative kinase 1	PINK1	hs00260868
Snail family zinc finger 2	SNAI2	hs00950344
Succinate dehydrogenase complex, subunit A, flavoprotein (Fp)	SDHA	hs00417200
Succinate dehydrogenase complex, subunit B, iron sulfur (Ip)	SDHB	hs01042482
Succinate dehydrogenase complex, subunit C, integral membrane protein	SDHC	hs01698067
Succinate dehydrogenase complex subunit D	SDHD	hs00829723
Vimentin	VIM	hs00185584

B

Antibody	Catalog number	Company
Anti-DRP1 antibody	ab56788	Abcam
Anti-N Cadherin antibody	ab18203	Abcam
Anti-SDHC antibody	ab155999	Abcam
Anti-SDHA antibody	ab14715	Abcam
Anti-SDHB antibody	ab14714	Abcam
Anti-Vimentin antibody	ab8978	Abcam
Anti-Vimentin antibody	ab92547	Abcam
E-Cadherin	3195	Cell Signaling
E-Cadherin	ab40772	Abcam
E-Cadherin	14472	Cell Signaling
Goat anti-Mouse IgG (H+L) Cross-Adsorbed Secondary Antibody, Alexa Fluor 594	a11005	Thermo Fisher
Goat anti-Rabbit IgG (H+L) Cross-Adsorbed Secondary Antibody, Alexa Fluor 647	a21244	Thermo Fisher
PGC1 alpha	sc-13067	Santa Cruz
Purified Mouse Anti-OPA1	612606	BD Transduction Laboratories ™
Tom20 Antibody (FL-145)	sc-11415	Santa Cruz Biotech,
HIF1a Antibody	#3716	Cell Signaling

C

Dye	Catalog number	Company
Alexa Fluor™ 555 Phalloidin	A34055	Thermo Fisher
Hoechst	33342	Thermo Fisher
ProLong™ Diamond Antifade Mountant with DAPI	P36962	Thermo Fisher
Pyronin Y	213519	Sigma

Table S3: (A) Probes used in experiments presented. (B) Antibodies used in experiments presented. (C) Probes and dyes used in experiments presented.
